# Rapid Assessment of Honey Quality and Authenticity via Electrochemical Determination of L-Proline and 5-Hydroxymethyl-2-Furfural Using Modified Pencil Graphite Sensors

**DOI:** 10.3390/foods15183325

**Published:** 2026-09-19

**Authors:** Berrin Gürler Akyüz, İzzet Koçak

**Affiliations:** Department of Basic Pharmaceutical Sciences, Faculty of Pharmacy, Zonguldak Bülent Ecevit University, Ibn-i Sina Campus, Esenköy Kozlu, Zonguldak 67600, Türkiye; izzetkocak@beun.edu.tr

**Keywords:** honey quality, food authenticity, L-proline, HMF, molecularly imprinted polymers, electrochemical sensor, PGE

## Abstract

Rapid and reliable quality control of honey is crucial for ensuring food safety and preventing food fraud. In this study, novel sensitive and selective molecularly imprinted polymer (MIP) sensors were developed for the direct and sequential determination of L-proline (L-Pro) and 5-hydroxymethyl-2-furfural (HMF). The sensors were fabricated via the electropolymerization of pyrrole (Py) on a pencil graphite electrode (PGE) surface, using L-Pro and HMF as template molecules. The linear working range for L-Pro and HMF using differential pulse voltammetry (DPV) is 10–1600 µM, with limits of detection determined as 5.39 µM and 4.81 µM, respectively. The MIP-PPy/PGEs demonstrated high selectivity against a 25-fold excess of structural analogues (response <25%). The sensors were highly reproducible (RSD 3.20–3.22%, *n* = 5) and repeatable (RSD 1.97–2.27%, *n* = 5), maintaining 81.5% stability over 15 days. The developed sensors successfully quantified native L-Pro and HMF levels in six real honey samples, monitoring thermal-stress-induced changes at 70 and 90 °C. Under increasing thermal stress, a consistent inverse relationship was observed, with L-Pro decreasing as HMF increased. The electrochemical results correlated strongly with standard UV and HPLC methods (RSD < 3%), offering a rapid, cost-effective, practical alternative for routine honey quality and authenticity control.

## 1. Introduction

L-proline (L-Pro) is a biologically significant amino acid in the human body and serves as a prominent quality indicator in various food matrices, most notably in honey [1,2,3]. In the context of honey quality control, L-Pro is recognized as the most abundant free amino acid, accounting for more than 50–80% of the total amino acid content, and is primarily introduced through honeybee salivary secretions during nectar processing [3,4]. Its quantification is highly critical because a decline in its standard levels serves as a direct indicator of premature harvesting, inadequate maturity, or industrial sugar syrup adulteration [1,5]. In international food regulations established by the Codex Alimentarius and the European Union (EU), a minimum proline concentration of 180 mg/kg is strictly required to qualify the authenticity and ripeness of the product, while a baseline above 300 mg/kg is widely accepted in quality control laboratories as a benchmark for premium unadulterated honey and its antioxidant properties [5,6]. Therefore, monitoring L-Pro levels represents a fundamental requirement for comprehensive food safety and authenticity assessment [4,5].

On the other hand, HMF is a cyclic aldehyde formed as a break-down product of hexose sugars via acid-catalyzed dehydration or the Maillard reaction during heating or prolonged storage [7,8]. Consequently, the HMF level contained in honey is regarded as a freshness indicator. While it is virtually non-existent in fresh and well-preserved honeys, it shows a natural increase over time; this is because it is triggered particularly by factors such as heat, sunlight, and metal ions that accelerate the course of the Maillard reaction [9]. In international honey trade regulations, the maximum tolerated HMF concentration is generally set below 40 mg/kg (and up to 80 mg/kg for honeys originating from tropical regions) as a prominent quality and freshness marker to evaluate the processing history and storage conditions of the product [5,8,10,11]. Consequently, the sequential analysis of both markers—where L-Pro serves as an indicator of authenticity and maturity, and HMF acts as a key parameter for heat stress and degradation—represents an integral and holistic aspect of comprehensive honey quality evaluation.

Conventional analytical techniques such as UV–Visible spectrometry [2,6,12], HPLC (High-performance liquid chromatography) [2,10], and GC-MS (Gas chromatography-mass spectrometry) [10] are quite accurate and efficient but are not suited for rapid on-site testing because of the required extensive sample preparation and expertise of the analyst. In contrast, electrochemical methods present a very attractive alternative with the promise of ease, speed, low-cost, and sensitivity [11,13]. Recent advancements in voltammetry have demonstrated the significant potential of this technique for food analysis. New modifications to electrodes have successfully enabled direct selective detection of complex analytes in challenging matrices [1,5,14].

In the past few years, the field of voltammetry, especially cyclic (CV), differential pulse (DPV), and square wave voltammetry (SWV), has gained importance for the determination of important biological and chemical analytes [15]. In recent studies, the utility of modern voltammetric sensors in applications such as food quality measurement, environmental studies, and biological applications, especially their modification on electrode surfaces and signal amplifications, was discussed [16,17,18,19]. In addition, approaches using nanomaterials and functional polymers to improve the electrochemical properties, particularly electron transfer kinetics and signal sensitivity, for the selective determination of analytes, especially in challenging media [20,21].

Among these approaches, MIPs have attracted significant interest because they can offer artificial recognition sites with high selectivity toward target molecules. The properties of MIPs afford tough synthetic polymers with molecular recognition characteristics similar to those of biological receptors [22,23]. Such MIPs, when incorporated into voltammetric sensors, allow selective analyte binding on the electrode surface, hence enhancing the sensitivity and discrimination against interfering species [24,25]. On account of their excellent electrical conductivity, ease of electropolymerization, chemical stability, and strong adhesion to electrode substrates, conducting polymers are widely used as MIP matrices; among them, PPy (polypyrrole) is the most extensively used for molecular imprinting [26]. Moreover, PPy films also act as protection layers by preventing electrode fouling, hence enhancing long-term stability in repetitive measurements [27].

The challenge in the development of selective electrochemical sensors for individual honey compounds, however, remains. Though there have been electrochemical sensors designed for the individual compounds of honey, such as L-Pro [24,28,29] and HMF [30,31,32], the achievement of high selectivity in a real sample such as honey poses a major limitation. To overcome this limitation in selectivity, the use of MIPs has proven to be a promising method to provide artificial recognition sites on the sensor surface. The technique involves the electropolymerization of a functional monomer such as pyrrole in the presence of a target molecule referred to as a template. This leads to the formation of a polymeric film containing shape-receptor sites. The removal of the template finally provides a means of specific rebinding, which improves selectivity to a remarkable extent [33,34]. The method not only simplifies the sensor’s design, but the polymeric layer also ensures a close contact with the transducer, thus maximizing the transduction of the analytical signal [35,36].

This technique has also been successfully employed by our group and other researchers for the analysis of various samples like antibiotics present in biologic fluids [4,37]. It is noteworthy to mention here that recent studies have also revealed the efficacy of PPy-based MIPs and the application of voltammetric methods for the sensitive analysis of amino acids and food contamination like HMF, establishing the maturity of the technique being used for the analysis of food samples [1,14,30,35,38]. For example, Francisco et al. described the sensitive electropolymerized MIP for HMF analysis [24], and Santos et al. also employed the optimized voltammetric method for HMF on the screen-printed electrode, establishing the trend for the easy application of the electrochemical method for the analysis of samples [28].

Nevertheless, there is still a substantial gap for a single, low-cost sensing platform capable of detecting both of these important indicators of honey quality. Moreover, a comprehensive study evaluating the effects of fabrication parameters—such as polymerization kinetics and template removal efficacy—on the analytical performance of PPy-based MIPs on disposable electrodes has not been conducted for this specific application. The objective of this study was to develop imprinted PPy/PGE electrochemical sensors, one for L-Pro and one for HMF, based on a common low-cost disposable electrode design, and to evaluate their analytical performance for complementary monitoring of honey compositional and heat-treatment indicators. In this regard, herein, we developed and optimized novel MIP-PPy modified PGE sensors for the sequential determination of L-Pro and HMF. Ultimately, this work provides a rapid, green, and practical electrochemical alternative to conventional chromatographic techniques, establishing a robust tool for routine food quality assurance and authenticity testing in the honey industry.

## 2. Materials and Methods

### 2.1. Instrument

Electrochemical measurements; CV, DPV and Amperometry (Amp) were performed by Autolab PGSTAT Potentiostat/Galvanostat (Metrohm Autolab, Utrecht, The Netherlands) with NOVA 2.1.4 software. The pH was measured using a SevenMulti pH/ion meter (Mettler-Toledo, Graifensee, Switzerland) with a glass electrode and an ISOLAB magnetic stirrer (ISOLAB Laborgeräte GmbH, Wertheim, Germany) and Elmasonic P ultrasonic bath (Elma Schmidbauer GmbH, Singen, Germany) were used to mix the solutions. The morphological characteristics of sensing platforms were investigated using the Field Emission Gun Scanning Electron Microscope (FEG-SEM, Quanta 450, Eindhoven, The Netherlands) technique. Fourier transform infrared (FT-IR) spectra in the range of 4000–500 cm^−1^ were recorded using KBr pellets on a Perkin Elmer FT-IR Spectra (Waltham, MA, USA). HPLC measurements were also carried out using Shimadzu SPD-20AV LC system along with a UV detector (Kyoto, Japan).

### 2.2. Chemicals and Reagents

L-proline (99.0%, Central Drug House, India); 4-aminobenzoik acid (99.0%), lithium perchlorate (98.0%), (Alfa Aesar, USA); acetonitrile (≥99.9%), sodium hydroxide (≥99.0%), 5-(Hydroxymethyl)-2-furfural, pyrrole (≥97%), 5-Methylfurfural (≥99.0%) (Merck, Germany); acetic acid (99.7–100%), KH_2_PO_4_, K_2_HPO_4_ (99.0%, Emir Chemistry), boric acid (99.0%, Galenik Pharma), KCl (99.0%, Zag Chemistry) (Türkiye); nicotinic acid (99.0%, TCI, Japan), ultra-pure deionized water (Human Power purification system, South Korea); phosphoric acid (85.0–88.0%), L-Tyrosine (≥98.0%), L-Alanine (≥98.0%), L-Phenylalanine (≥98.0%), glucose (≥99.5%), fructose (≥99.0%), sucrose (≥99.5%), lactose (≥98.0%), dopamine (≥98.0%), L-cysteine (≥97.0%), L-glutamic acid (≥99.0%) and caffeine (≥98.5%) (Sigma Aldrich, USA); Glycine (≥99.0%), gallic acid(≥98.0%), uric acid (≥98.0%), ascorbic acid (≥99.0%) were purchased from Fisher Scientific (USA).

### 2.3. Electrochemical Measurements

A conventional three-electrode assembly was utilized to execute the electrochemical characterizations and quantitative assays. Within this framework, the molecularly im-printed and non-imprinted PPy-PGE (Graphite leads, Tombow, HB, 0.7 mm diameter, Japan) [30,33,34] served as the working electrode, complemented by a platinum wire acting as the auxiliary counter electrode. All measured potentials were referenced to an Ag|AgCl|3 M KCl electrode system. The analytical responses for both L-Pro and HMF were recorded using the DPV method over a potential range of 0 to 1.7 V and 0 to –1.5 V, respectively. The pulse modulation was maintained at an amplitude of 25 mV, while the scan rate was fixed at 100 mV/s.

### 2.4. Optimization of Parameters

#### 2.4.1. Monomer Type Selection

In order to select the appropriate monomer, 3 different monomers were used: nicotinic acid (NA), para-amino benzoic acid (PABA) [24] and Py [30,33,34,37]. For each monomer, MIP and NIP (non-imprinted polymer) electrodes were prepared separately with and without L-Pro. However, in the electrodes prepared with NA and PABA coated at low cycle numbers, it was observed that the polymers started to be removed from the PGE surface in the target molecule removal processes, so the study was continued with Py.

#### 2.4.2. Supporting Electrolyte Selection

LiClO_4_ [30,37] and KCl [39] were tested as supporting electrolytes. KCl produced substantially higher oxidation and reduction peak currents compared with LiClO_4_, and was therefore selected as the supporting electrolyte for all subsequent experiments.

#### 2.4.3. Buffer Selection

Britton–Robinson buffer (BRT) [28] and phosphate buffer (PBS) at different pH values were used to determine the optimum buffer medium. After several pH tests, PBS yielded higher and more clearly resolved oxidation and reduction peaks and was therefore selected as the working buffer, with pH 7.4 identified as optimal [6,24,37].

### 2.5. MIP and NIP Sensor Preparation Procedures on PGE

MIPs are artificial receptors made by mixing a template molecule and functional monomers in a solvent to form a complex via covalent or non-covalent bonds. After polymerization, the template is removed, leaving behind custom cavities that selectively capture the target analyte based on its shape, size, and chemical properties [40]. These customized cavities function as selective recognition sites, enabling the isolation of the target molecule from complex mixtures. The strength and specificity of the interaction depend on the configuration and nature of the functional groups within the cavities, rendering MIPs highly efficient for diverse sensing technologies that demand strict selectivity [22].

In this work the PGEs were prepared by cutting the pencil graphite leads into 3 cm long sticks [30,33,34]. Prior to experimentation, PGEs were meticulously washed with a solution of water and acetonitrile, a process employed to ensure the removal of any extraneous impurities. Thereafter, the PGEs were dried at room temperature. To prepare the PGE-MIP and PGE-NIP modified electrodes, each PGE was immersed in the respective polymerization solution (with the target molecule added for MIP). Subsequently, electropolymerization of Py onto the pencil graphite surface was performed by CV between 0 and 2 V at a scan rate of 10–1000 mV/s. Following polymerization, there are various methods to remove the target molecule from the electrode surface. In this study, two different techniques were evaluated: mechanical stirring [24] and over-oxidation via CV [22,30,33,34]. First, the imprinted PGE-MIP-PPy films were over-oxidized by CV at a scan rate of 50 mV/s from +0.80 to +1.20 V [30,33,34] 10, 15 and 20 cycles in 0.01, 0.05 and 0.1 M NaOH solutions. This over-oxidation process simultaneously oxidized the polymer matrix and extracted L-pro form the PGE-MIP-PPy films. Second, PGE-MIP-PPy films were immersed in 0.01, 0.05 and 0.1 M NaOH solutions for 3, 4 or 5 min, under both stirring [24] and static conditions.

#### Over-Oxidation of PGE-MIP-PPy and PGE-NIP-PPy Films

Over-oxidation is a post-polymerization modification method that incorporates oxygen groups into conducting polymers like PPy to create selective binding cavities and enhance interaction with target molecules. This process improves molecular recognition by adding carboxylic groups, enhances the functionalization of the polymer matrix, and facilitates template removal, thereby boosting the selectivity and regeneration of e-MIP-based sensors [22].

Over-oxidation process was carried out by CV, taking 10, 15 and 20 cycles of the PGE-MIP-PPy and PGE-NIP-PPy electrodes in 0.01, 0.05 and 0.1 M NaOH solutions [30,33,34]. A sample of 0.1 M NaOH medium [24] was selected as the most suitable medium for removing the target molecule by means of an over-oxidation process.

### 2.6. L-Pro and HMF Determination in Real Samples by DPV

The prepared PGE-MIP-PPy sensors were used to quantify L-Pro in a total of 6 different honey samples. Three of the samples were flower honey obtained from commercial markets. Two of the samples were traditional flower honey and chestnut honey obtained from local producers. The final sample was sunflower honey obtained from the producer.

Initially, the sensors were prepared, and the L-Pro and HMF contents of the honey samples were measured at room temperature without any treatment. Next, 5 g honey samples were incubated in a water bath at 70 °C for 2 h. After cooling to room temperature, their L-Pro and HMF contents were determined via DPV. Subsequently, the samples were kept in a water bath at 90 °C for 2 h, and the same procedures were repeated. 

It is also essential to mention that in the case of honey extracts whose initial DPV signal indicated an L-Pro concentration beyond the upper limit of the linear calibration range, a precise dilution with the supporting 0.1 M PBS, pH 7.4 was carried out in order to fall within the validated linear dynamic range, while the final concentration was calculated by applying the corresponding dilution factor.

### 2.7. HPLC and UV Analysis of Real Honey Samples

HPLC was employed as a reference technique in the validation of the electrochemical data obtained in HMF. The HPLC analysis was conducted on a Shimadzu SPD-20AV HPLC system along with a UV detector. The analytical column used in the experiment is a reversed-phase column (C18 column) at ambient temperatures.

The mobile phase was water:methanol (90:10, *v*/*v*) and was delivered isocratically at a flow rate of 1.0 mL min^−1^. The injection volume was 20 µL, and the detection wavelength was 284 nm. The honey samples used for analysis were collected from both local markets and direct producers, stored at room temperature, and in the dark until the time of analysis. Before carrying out HPLC measurements, honey samples were handled as previously mentioned and filtered using a 0.22 µm membrane filter for removal of suspended particulates prior to injection. The amount of HMF was determined by using external calibration curves. The samples were injected in triplicate.

In order to validate the accuracy of the newly developed L-Pro sensor, the amount of proline in the honey samples was additionally determined via UV method. Namely, 5 g of honey was weighed with a precision of 0.1 mg, and was dissolved in 50 mL distilled water and diluted up to the mark in 100 mL volumetric flask with distilled water. In each series of tests, 0.5 mL of honey solution, 0.5 mL of distilled water (as blank) and 0.5 mL of proline standard solution were pipetted separately into screw-capped test tubes, where the last one was tested in at least three parallel tests. Then, to each of the tubes, 1 mL of freshly prepared ninhydrin reagent (1.5 g in 50 mL of ethylene glycol monomethyl ether) and 1 mL of formic acid (98–100%, m/m) were added. The tubes were placed in the water bath at 70 °C for 30 min, aiming to facilitate the colour formation. After 30 min, 5 mL of 50% (*v*/*v*) 2-propanol was added to each tube, aiming to stop the reaction. The tubes were then quickly capped and cooled to room temperature. Following an additional 45 min incubation at room temperature, the colour was stabilized, and the absorbance was measured at 510 nm.

## 3. Results

### 3.1. pH Effect

The choice of the buffer medium and the optimization of the buffer pH are highly critical parameters, as they directly dictate the protonation state of the target molecules (L-Pro and HMF), the stability of the electropolymerized PPy matrix, and the subsequent electron transfer kinetics. The electrochemical performance of the modified PGE toward the target analytes was investigated within a pH range of 2.0 to 9.0, utilizing two distinct buffering systems: Britton–Robinson buffer (BRT) [28] and phosphate buffer solution (PBS) [6,10,22,24,25,37].

In this study, the specific pH and buffer conditions were systematically selected based on a combination of preliminary experimental evaluations and established electroanalytical literature [22,37]. PPy films exhibit optimal conductivity and structural stability in weakly acidic to neutral aqueous environments, whereas highly alkaline media trigger the irreversible deprotonation and over-oxidation of the polymer backbone, leading to a significant loss in electroactivity [32]. Furthermore, under the optimized buffer conditions, the target analytes retain their ideal structural charge compatibility with the molecularly imprinted cavities. This synergistic environment maximizes the selective rebinding interaction towards both L-Pro and HMF while concurrently suppressing capacitive background currents, thereby ensuring the highest analytical sensitivity and reproducibility for honey matrix screening [25,37].

**Figure 1:**
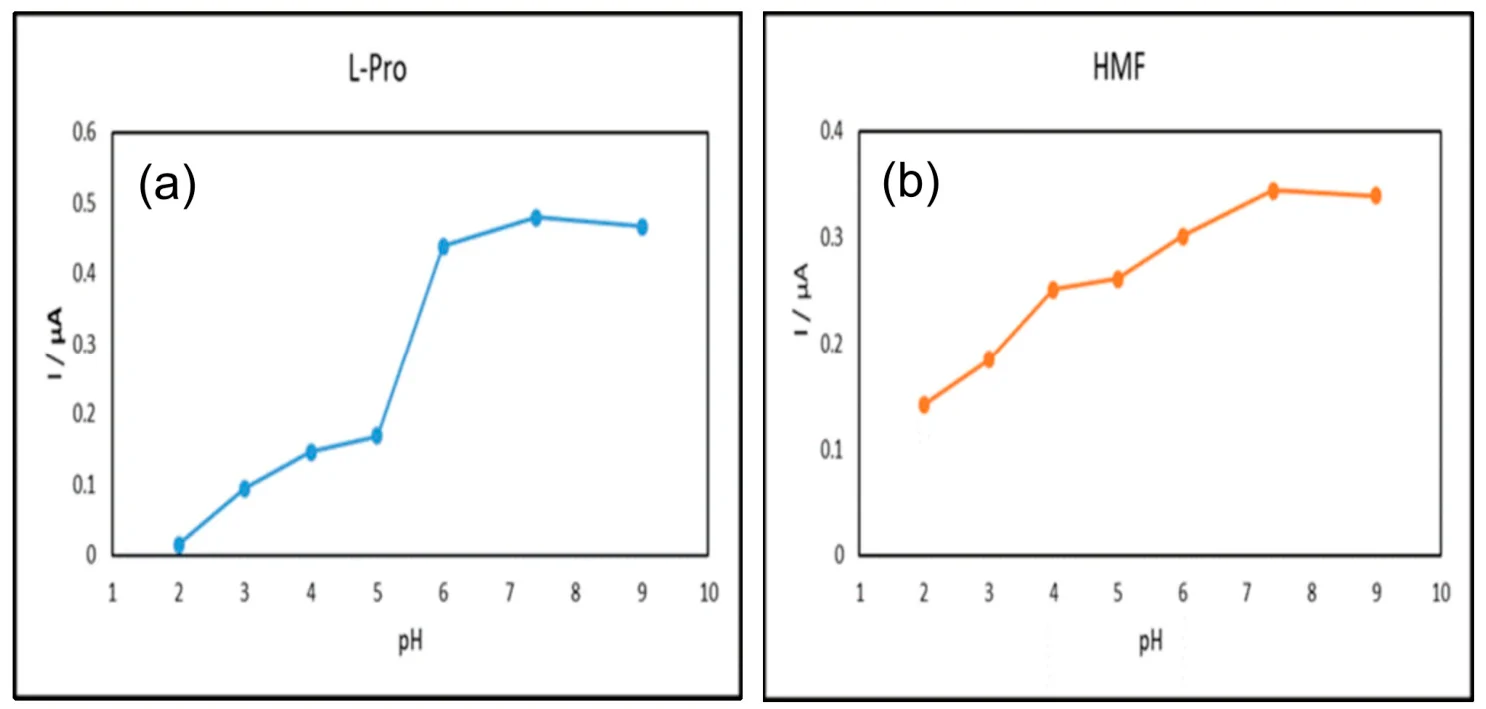
The effect of pH (2–9) of the oxidation and reduction peak current response of (**a**) L-Pro and (**b**) HMF in PBS using a PGE-MIP-PPy electrode.

Based on the optimal current values and peaks obtained from buffer solution experiments conducted at different pH values, as well as data from studies in the literature, it was determined that a phosphate buffer at pH 7.4 [6,22,24] is the most suitable operating environment. The relevant results are shown in Figure 1a,b.

### 3.2. Template Molecule Removal

**Figure 2:**
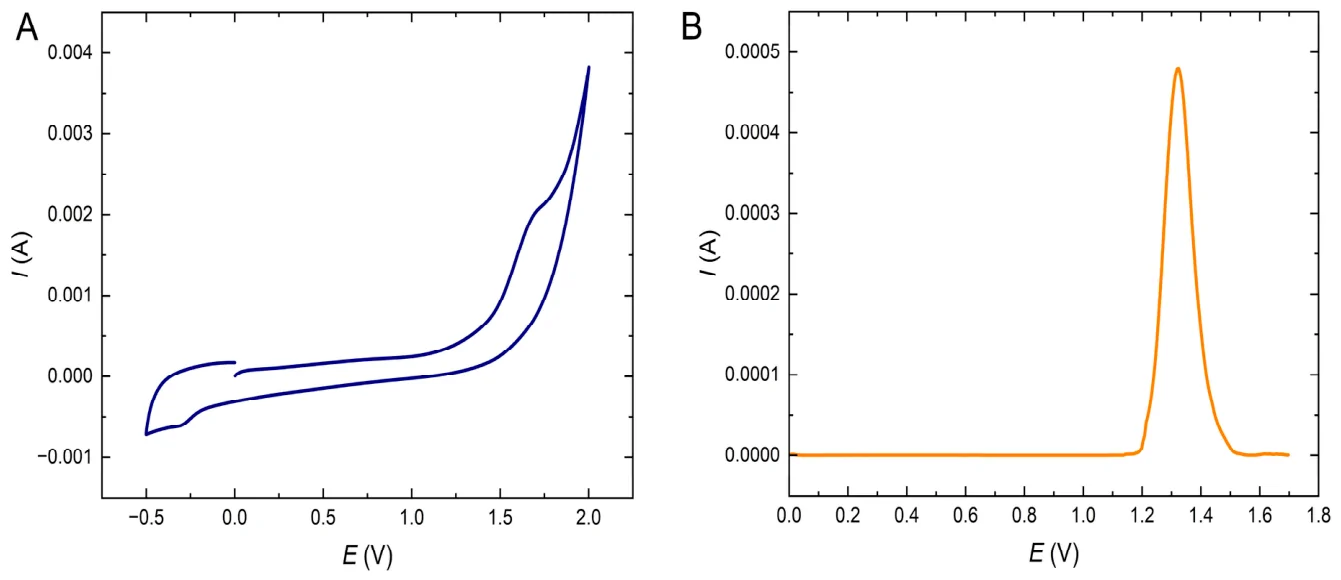
Voltammograms of L-Pro in 0.1 M NaOH, with PGE-MIP-PPy by (**A**) CV recorded at 100 mV s^−1^ (**B**) DPV recorded at modulation amplitude 25 mV s^−1^ and scan rate 10 mV s^−1^.

In order to confirm the removal of the target molecule, the electrodes were rinsed, dried under a stream of nitrogen and placed in a cell containing NaOH solution serving as the stripping solution; current intensity was then measured using the CV and DPV method. Obtained results revealed that the over-oxidation method yielded better results than the stirring method in removing the target molecule from the polymeric structure. It was also observed that for the electrodes subjected to 15 cycles of over-oxidation in 0.1 M NaOH solution, this was the best electrolyte medium for over-oxidation and target molecule removal. In this solution, L-Pro was determined by DPV in the range between 0 and +1.7 V with covered PGE. The voltammograms in Figure 2A,B show the template molecule extracted from the polymer and transferred to a NaOH solution.

### 3.3. Electrochemical Behavior of L-Pro on PGE-MIP-PPy

**Figure 3:**
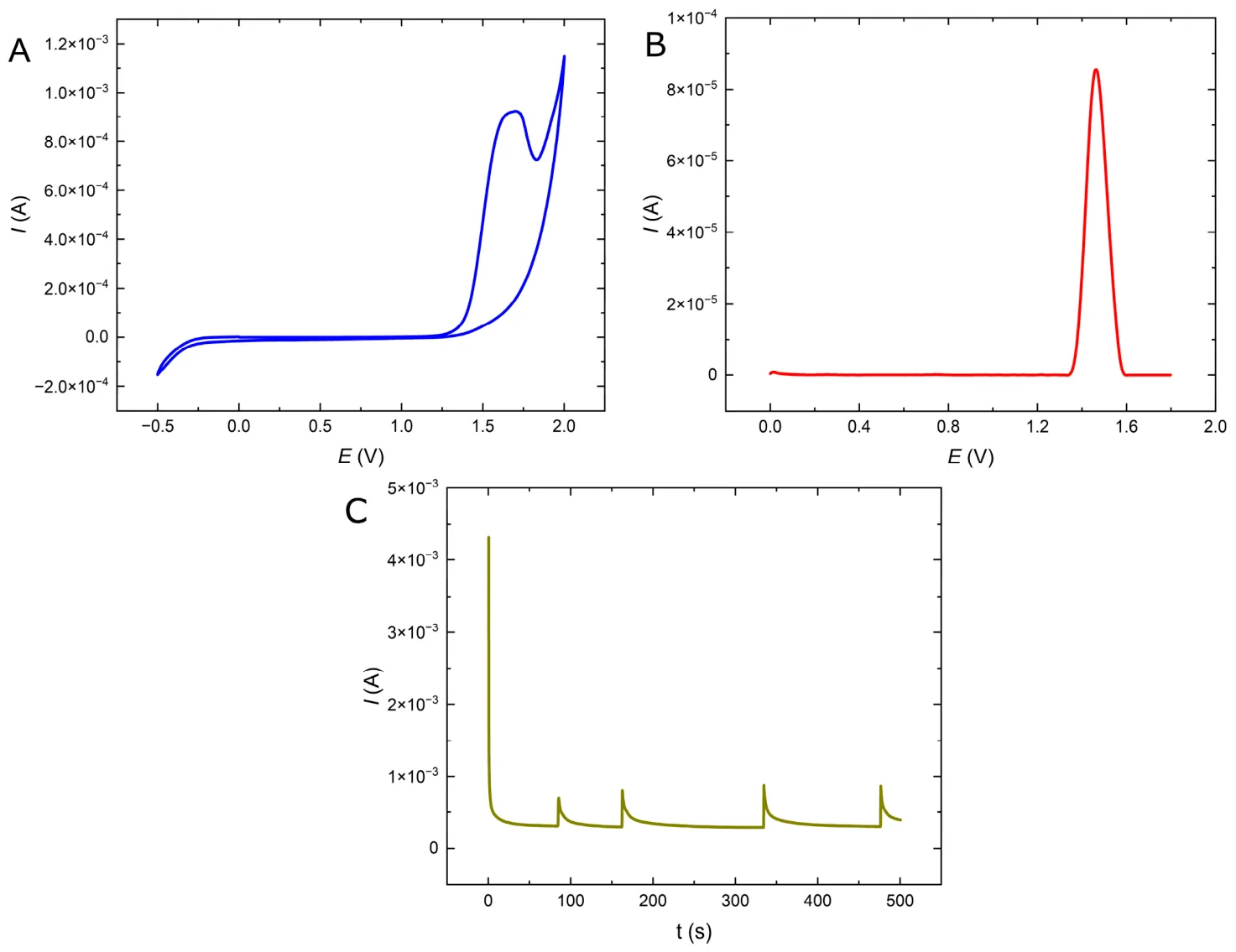
The voltammograms of the electrochemical behavior of L-Pro in 0.1 M PBS at pH 7.4, (**A**) CV, (**B**) DPV and (**C**) Amp.

Different methods have been used to determine the electrochemical behavior of L-pro and PGE-MIP-PPy. These are CV, DPV and Amp, as illustrated in Figure 3A–C, the voltammograms corresponding to the methods employed are presented in the order of their utilization. By all three methods, the oxidation voltage of 50 µM L-Pro was determined to be around +1.5 V at pH 7.4 in 0.1 M PBS. Even though L-Pro is an amino acid that is typically not electrochemically active on carbon electrodes, the oxidation peak observed at around +1.5 V in the L-Pro solution represents a mediated oxidation reaction catalyzed by the overoxi-PPy matrix. The over-oxidized layer produces a surface rich in carbonyl and/or carboxyl groups that can serve as redox sites. Consequently, the L-proline molecularly imprinted into the matrix will be oxidized at these redox sites, producing the characteristic anodic current [41]. This is also supported by the fact that the observed +1.5 V prominently increased on the MIP sensor compared to the NIP sensor, clearly indicating that the observed +1.5 V peak indeed reflects the characteristic interaction signal between L-Pro and the overoxi-PPy matrix and does not represent the oxidation signal of L-Pro or overoxi-PPy itself. We conducted a CV experiment (with varying scan rates from 10 to 500 mV/s) on the optimized PGE-MIP-PPy sensor in a solution of fixed concentration of L-Pro or HMF. As seen in Figure S1, for each of the substances, a graph of the anodic peak current (I_pa_) for L-Pro and the cathodic peak current (I_pc_) for HMF, versus the square root of the scan rate, provided a straight line with high correlation factor (R^2^ > 0.99). Linear variation of the peak current with respect to the square root of the scan rate, instead of variation with scan rate itself, confirms that the electrochemical reaction on the surface of PGE-MIP-PPy follows diffusion control, in accordance with Randles–Ševčík equation. This is because a freely diffusing electroactive species shows such behavior. This is contrary to a redox couple which is confined to the surface or controlled by adsorption, where the peak current varies linearly with υ itself. In our case, the diffusion control for both the anodic (L-Pro) and cathodic (HMF) peaks proves that the electrochemical process depends mainly on the transport of the analyte to the electrode surface, instead of the transport of a pre-adsorbed and surface-confined species. The prepared PGE-MIP-PPy electrodes were initially utilized for L-Pro and HMF calibration graph by standard addition method and subsequently for L-Pro and HMF determination by DPV in real honey samples. All the experiments were performed in triplicate.

### 3.4. Electropolymerization Cycle Effect

Electropolymerization enables precise control over the film’s thickness, structure, and the positioning of binding sites near the electrode surface [42]. Such features accelerate response times and deliver outstanding sensitivity. Additionally, electrosynthesis eliminates the need for organic solvents or auxiliary processing stages, rendering it a more eco-friendly and economically viable strategy [22].

The optimum CV cycles for the formation of the “sensing” layer of MIPs were detected from a series of experiments (5, 7, 9, 11, 15 cycles) in our study. It was observed that the number of polymerization cycles significantly affected the proline peak current. The use of a large number of cycles (>10) did not significantly increase the signal values and implied longer polymerization times, as well as resulting in a thicker polymer layer with a large number of cycles, making it difficult to successfully remove template molecules and create specific cavities. Conversely, with a small number of cycles (<5), a very thin layer of polymer is obtained; the polymer may be unstable and may not polymerize sufficiently around the target molecules to allow simultaneous polymerization. Therefore, the number of seven cycles was chosen as the optimum number [24,30,33,34].

**Figure 4:**
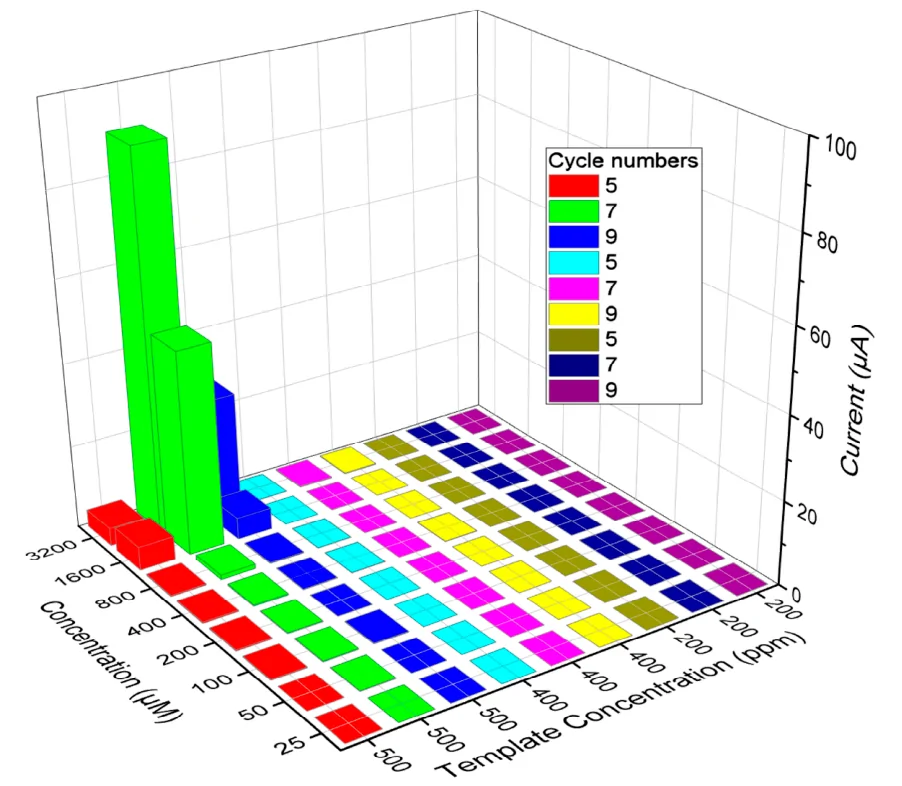
Influence of polymerization cycle number and template-to-monomer ratio on the sensor response (*n* = 3).

Figure 4 shows the evaluation of the target molecule-to-monomer ratio and polymerization cycle number, alongside the sensor’s response to concentration. It was observed that the electrodes imprinted with a 500 ppm template, coated with 7 cycles of polymeric film, and subjected to 15 cycles of overoxidation gave the highest current response.

### 3.5. Monomer/Template Ratio and Effect

The template-to-monomer ratio during electropolymerization represents a critical parameter that determines both the stability of the MIP and its selectivity for the target analyte [24].

During the optimization of the target molecule-to-monomer ratio, 200, 400, and 500 ppm L-Pro and 0.05 M and 0.1 M Py [30,33,34] were used. Our systematic screening revealed that the electrodes imprinted with a 500 ppm template molecule when 0.1 M Py was used as the monomer, coated with 7 cycles of polymeric film, and subjected to 15 cycles of overoxidation yielded the maximum peak current (Figure 4).

### 3.6. Influence of Electropolymerization Scan Rate on Sensor Performance

**Figure 5:**
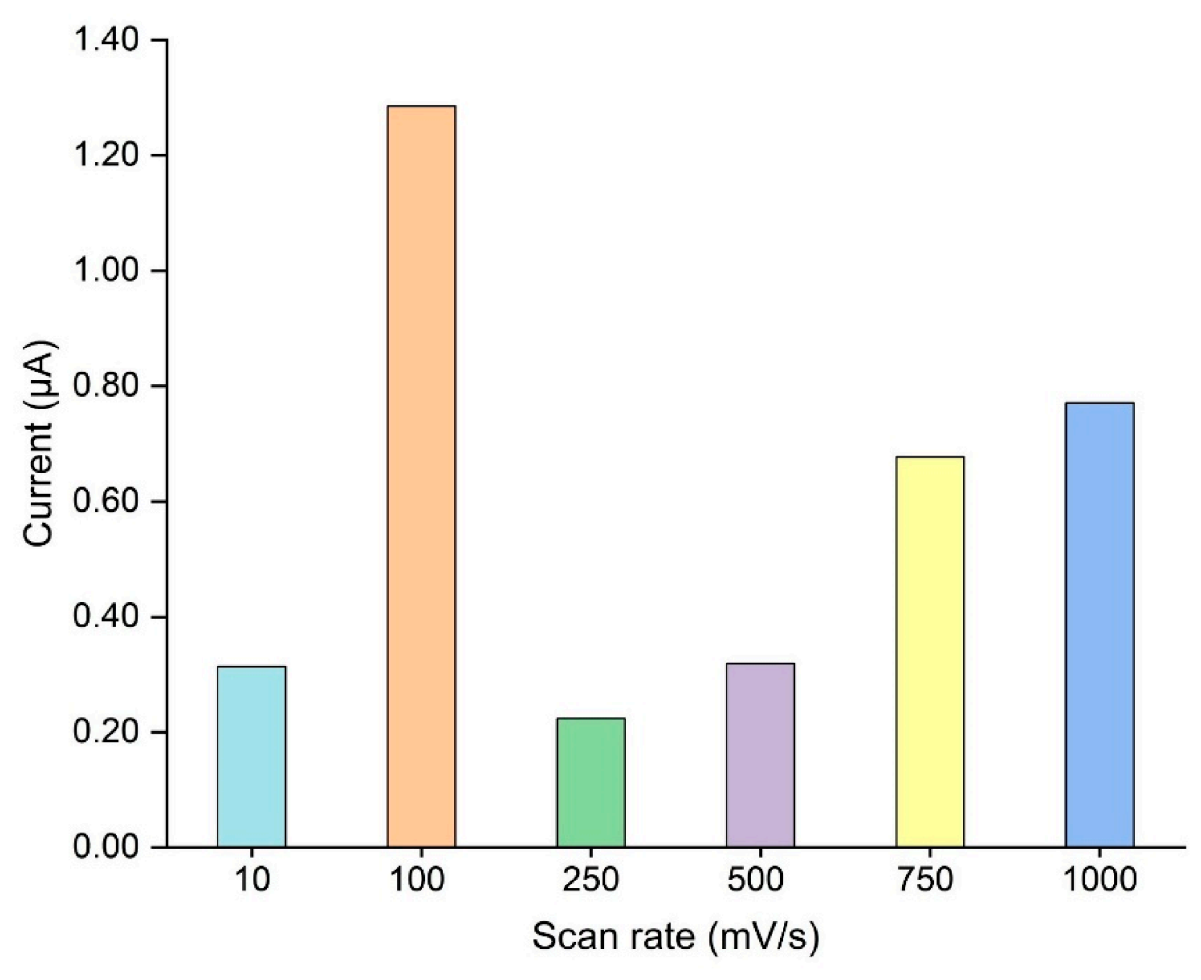
The electropolymerization scan rate determined based on the electrochemical response of the developed MIP sensor. The bar chart illustrates the comparative analytical peak currents recorded via DPV for functional polymer films synthesized across various deposition velocities (10, 100, 250, 500, 750, and 1000 mV/s), demonstrating 100 mV/s as the selected preparation condition for maximum sensitivity (*n* = 3).

To elucidate the impact of polymer matrix growth velocity on the subsequent electrochemical responsiveness, the functional PPy films were electrodeposited across a broad spectrum of scan rates (10, 100, 250, 500, 750, and 1000 mV/s), and their comparative sensing activities were systematically evaluated. As illustrated in Figure 5, the highest analytical sensitivity was achieved at an optimum scan rate of 100 mV/s. Structurally, the electropolymerization rate dictates the morphology, density, and thickness of the resulting molecularly imprinted framework [22,32]. Application of excessive scan rates (e.g., ≥250 mV/s) triggers accelerated polymer deposition, yielding disordered, highly irregular, and unstable architectures with poor adherence to the PGE surface [32,35]. Conversely, while exceptionally slow kinetics (10 mV/s) promote a compact and smooth film, it often restricts mass transfer due to dense polymer packing, which hinders effective template entrapment and subsequent removal [22,38]. Consequently, the selection of 100 mV/s represents an ideal equilibrium, offering a well-defined, robust, and moderately porous PPy network that ensures superior structural stability, enhanced adhesion, and maximal accessibility toward the target analytes [35,38].

### 3.7. Characterization of Sensing Surfaces

**Figure 6:**
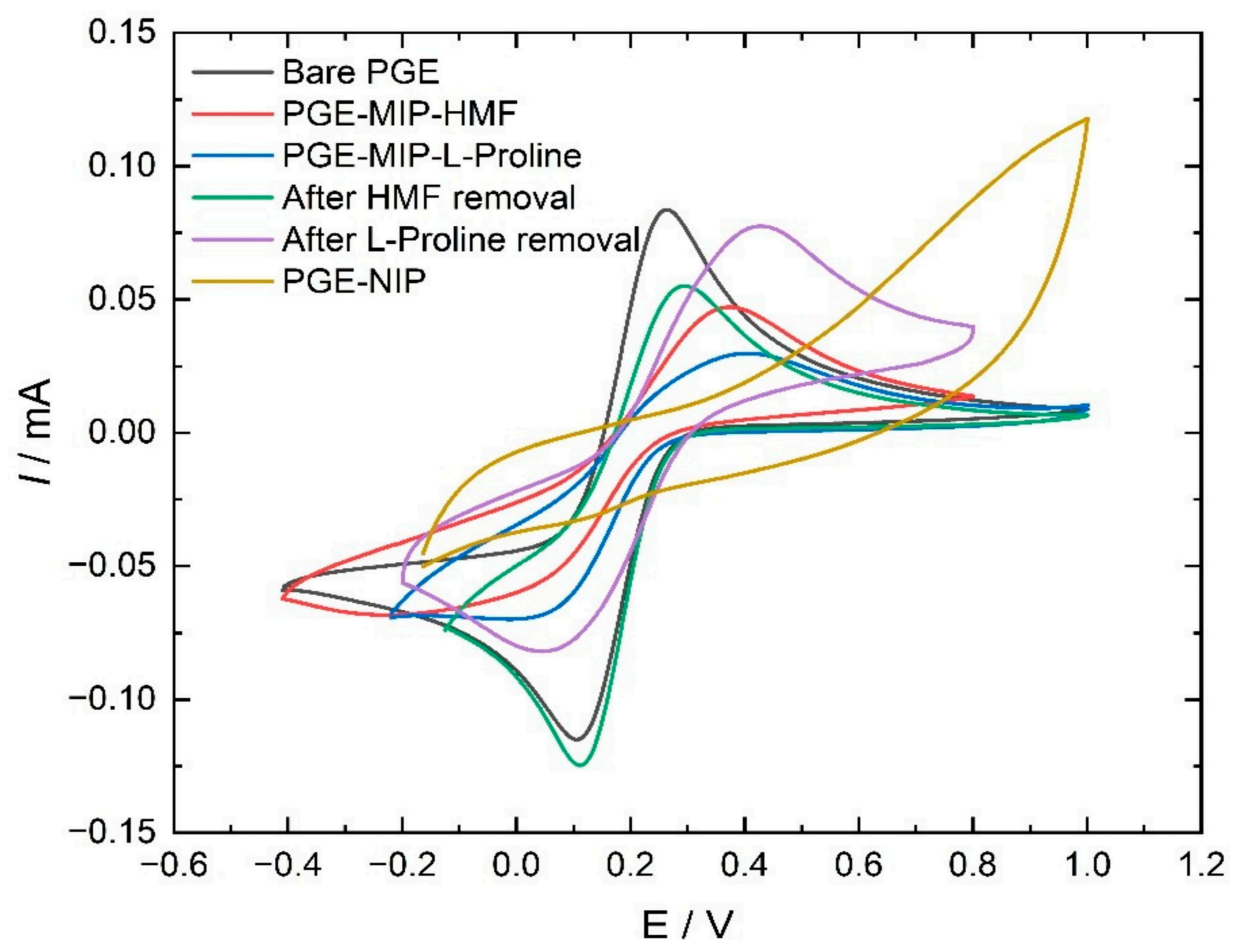
CV of bare PGE, PGE-NIP, PGE-MIP-HMF and PGE-MIP-L-Pro before and after template extraction in the presence of 5 mM [Fe(CN)_6_]^3−^/^4−^ at the 50 mV s^−1^.

Electrochemical surface characteristics of the sensing surfaces were investigated versus ferricyanide redox probe. Figure 6 presents the CVs of the bare PGE, PGE modified with MIPs for HMF and L-Pro (PGE-MIP-HMF and PGE-MIP-L-Pro), the corresponding electrodes after template removal, and the NIP electrode (PGE-NIP), recorded in a ferricyanide/ferrocyanide redox system. The bare PGE exhibits a well-defined, quasi-reversible behavior. This behavior is characteristic of a clean, electrochemically active carbon surface that facilitates efficient electron transfer to the solution-based redox probe. After the electrophoretic polymerization of Py in the presence of the template compounds (HMF and L-Pro), there can be seen a substantial decrease in the peak currents and a sharp increase in the peak-to-peak separation value (ΔE_p_) for both MIP electrodes compared to the PGE. This suggests that the PPy layer acts as a physical barrier against the diffusion of the negatively charged ions, namely [Fe(CN)_6_]^3−^/^4−^. However, the large peak-to-peak separation value suggests lower kinetics for the electron transfer process, indicating the deposited layer to be non-conducting and/or poorly conducting at the applied potential for polymerization. Finally, the decrease in peak currents indicates that the electrode surface has indeed been modified with the deposited polymer layer, entrapping the template compounds. After the removal of the templates, a distinct recovery of the redox peak currents for both MIPs can clearly be seen. The increase in peak current is ascribed to the formation of specific recognition sites within the PPy matrix that increase the permeability of the film to ions in the solution. The recovery of the electroactive areas serves as evidence for the successful removal of templates. On the other hand, comparing the complete suppression of the both anodic and cathodic peaks of ferricyanide at the NIP electrodes with the recovery of the redox reaction in the MIPs after the removal of the templates provides strong evidence for the electrochemical recognition process.

**Figure 7:**
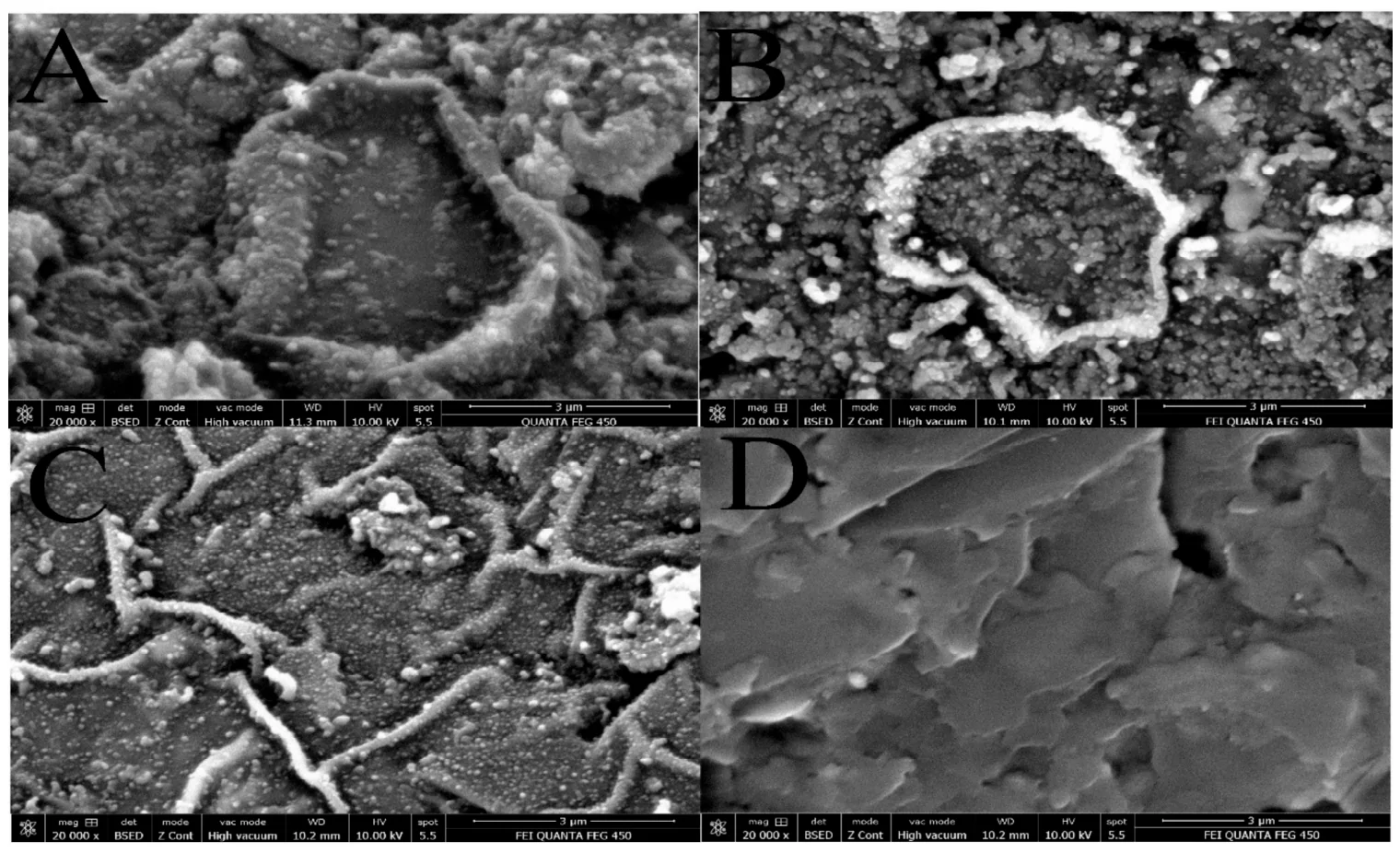
SEM pictures of (**A**) PGE-MIP-HMF (after template removal), (**B**) PGE-MIP-L-Pro (after template removal), (**C**) PGE-NIP, (**D**) PGE.

The surface morphology of plain PGE, PEG-NIP, PEG-MIP-HMF and PEG-MIP-L-Pro (after template removal) was studied using SEM to explain the effect of molecular imprinting on surface morphology. As seen in Figure 7A–D, the surface morphology of bare PGE appears very smooth with minimal surface details, indicative of a surface lacking a polymeric surface for molecular recognition. The surface morphology of the PGE-NIP appears rather homogenous with minimal surface roughness, indicative of a polymeric surface with no discernible cavities, which is characteristic of MIP surfaces created via electropolymerization after removal of templates. On the other hand, both the PGE-MIP-HMF and the PGE-MIP-L-Pro electrodes (without templates) exhibit very rough topographies. The porous structures, irregular cavities, and fissures on the MIP surfaces are easily identified and related to the removal of their respective template molecules from a polymer matrix. These holes are not found very much on the surface of the NIP and are proof that molecular imprinting was successful. Specifically, it is noted that the voids on the PGE-MIP-HMF surface appear relatively larger and sharper, as expected based on the larger dimensions of HMF, while on the PGE-MIP-L-Pro surface, smaller and more evenly sized voids are observed, as expected based on its smaller size and distinctive functional geometry. Additionally, the observed surface roughness along with imprinted voids on the electrode surfaces is expected to contribute to higher analyte accumulation at the electrode/solution interface, thus favoring electron transfer, thereby accounting for the observed higher voltammetric signals recorded using MIP electrodes compared to NIP and bare PGE electrodes. Such structural features offer clear evidence regarding the success of the imprinting process.

**Figure 8:**
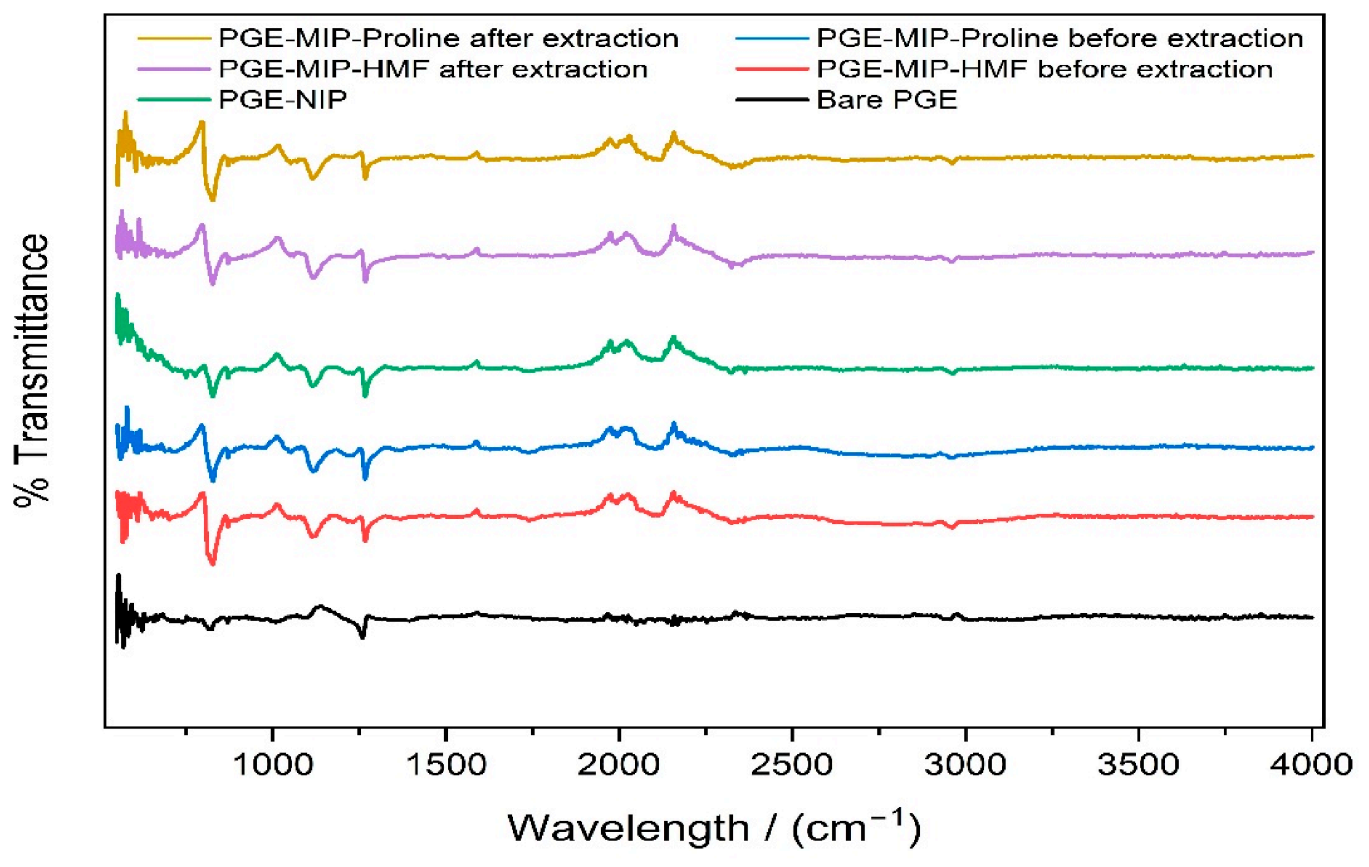
FT-IR spectra of plain PGE, PGE-MIP-HMF and PGE-MIP-L-Pro before extraction, PGE-MIP-HMF and PGE-MIP-L-Pro after extraction and PGE-NIP.

The FT-IR spectra of the bare PGE, PGE-NIP, PGE-MIP-L-Pro, and PGE-MIP-HMF electrodes, both before and after template removal, are presented in Figure 8. The bare PGE’s FT-IR spectrum shows some broad, featureless absorption peaks, ensuring the absence of the polymeric and organic functional groups on a bare PGE. The FT-IR spectra of all the polymer-coated electrodes (PGE-NIP and PGE-MIPs) give absorption peaks that correspond to the PPy. These are not found in the FT-IR spectrum of the bare PGE. The absorption at around 1580 cm^−1^ is attributed to the C=C and C-C bonds of the pyrrole rings. The broad band between 1000 and 1280 cm^−1^ includes the C-H in-plane deformation and the N-H bending vibrations. These consistent features ensure that the electropolymerization of the Py is successful on the PGE. Also, the presence of the HMF and the L-Pro is evidenced by the involvement of the carbonyl (C=O) and the hydroxyl (O-H) groups. The carbonyl stretch of the HMF is not observable at around 1670 cm^−1^ prior to the template removal. This strongly indicates that the C=O group of HMF or L-Pro are engaged in specific hydrogen-bonding interactions with the N–H groups of the PPy chain, resulting in a shift, broadening, and loss of intensity for this band. Concurrently, the O–H stretch of HMF or L-Pro are present at around 3000 cm^−1^. This is again a key indicator of successful imprinting; the O–H group is participating in a strong, cooperative hydrogen-bonding network with the polymer matrix, causing the band to become extremely broad and attenuated. The most significant observation is the lack of regeneration of sharp O–H or C=O bands after the extraction process. If the templates were merely physically adsorbed or trapped without specific interactions, their removal would leave behind unbound functional groups or show residual sharp bands. The fact that the spectra after extraction are virtually identical to the NIP confirms the complete removal of the template molecules. Furthermore, the disappearance of O–H/C=O features signifies that the hydrogen-bonding interactions responsible for the initial spectral broadening have been cleaved. The resulting spectral demonstrates that the extraction process successfully removes the template, leaving behind a cavity whose functional groups (the N–H of PPy) are now free and available for subsequent rebinding of the target analyte.

### 3.8. Sensitivity, Selectivity, Reproducibility and Stability Studies

**Figure 9:**
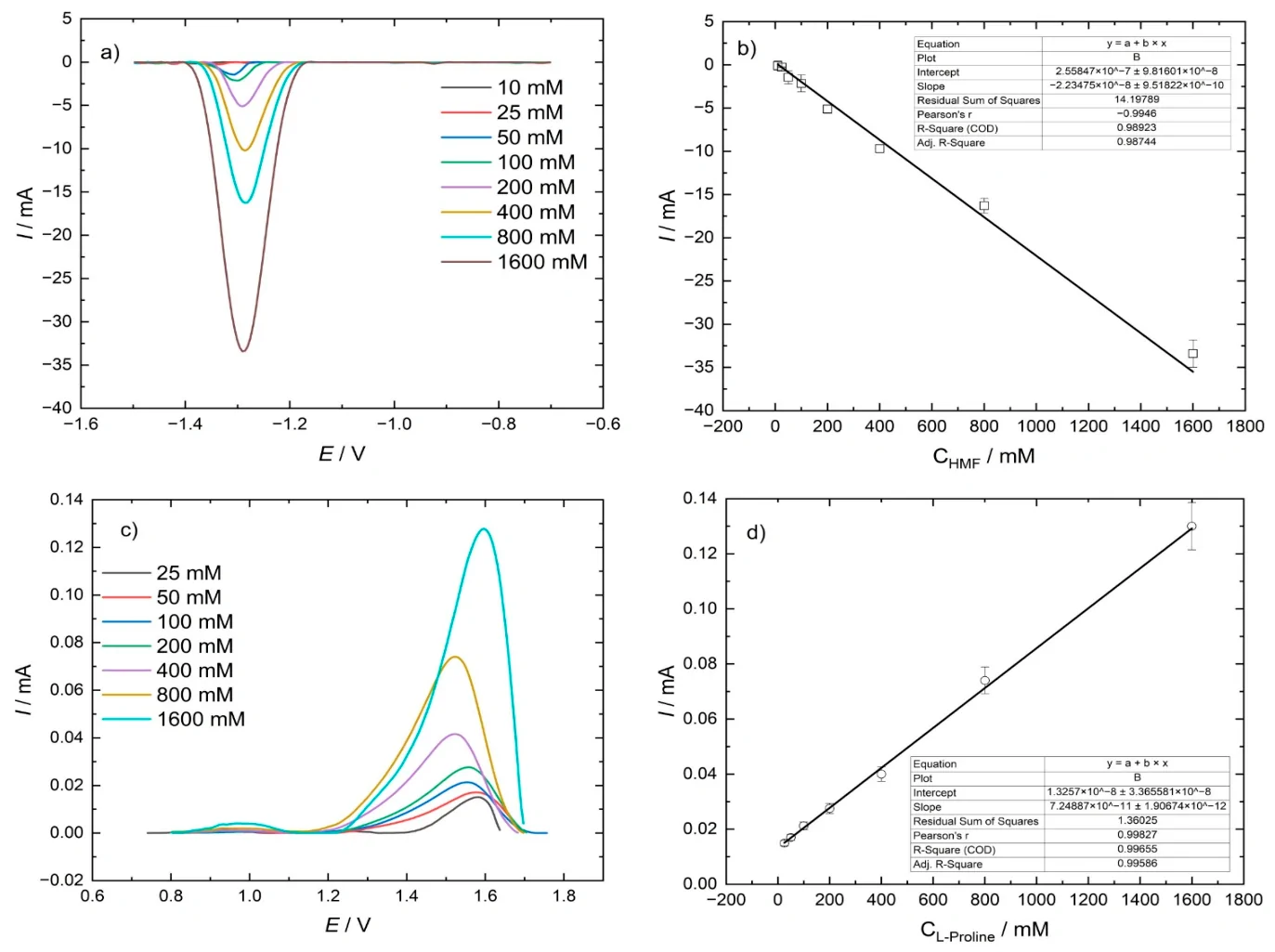
Differential pulse voltammograms of the reduction of HMF (**a**) and oxidation of L-Pro (**c**) at the PGE-MIP-PPy sensor at different concentrations in 0.1 M PBS at pH 7.4. Calibration graphs of (**b**) HMF (**d**) L-Pro.

The DP voltammograms for HMF and L-Pro, obtained using their respective MIP-modified PGEs, are illustrated in Figure 9a,c. For both platforms, a distinct calibration curve was recorded, showing the change in response as the concentration of the analyte increased.

In the case of the L-Pro sensor (Figure 9d), the result reveals a linear increase in the oxidation current with the increase in L-Pro concentration. This implies a successful mass transport process as well as a successful electron-transfer mechanism between the imprinted MIP-modified surface of the electrode and the L-Pro molecules. The linear response of the sensor indicates a successful binding affinity of the imprinted L-Pro molecules towards L-Pro molecules without any site saturation effect within the range of concentrations used. On the other hand, for the HMF sensor (Figure 9b), a linear decrease in the cathodic current was observed with an increase in the concentrations of HMF. This large linear range shown by HMF concentration further confirms the usefulness of the sensor in monitoring thermally induced HMF in concentrations typical of actual food samples. In this regard, the high linearity of both calibration plots underlines that the molecular imprinting strategy provides stable and reproducible recognition sites to allow for reliable quantitative determination of L-Pro and HMF. The distinct yet predictable current–concentration relationships further underline the versatility of the MIP-based electrochemical sensors for the detecting of chemically diverse analytes by means of tailored imprinting approaches.

The LOD and LOQ for both L-Pro and HMF were determined using the standard deviation of the blank signal (σ) and the slope of the corresponding calibration curves, employing the equations LOD = 3σ/slope and LOQ = 10σ/slope. The obtained results were for L-Pro 5.39 µM (6.21 mg kg^−1^) and 4.81 µM (6.07 mg kg^−1^) for HMF. The analytical performance of the developed method is compared with alternative literature studies for L-Pro and HMF determination in Table 1.

**Figure 10:**
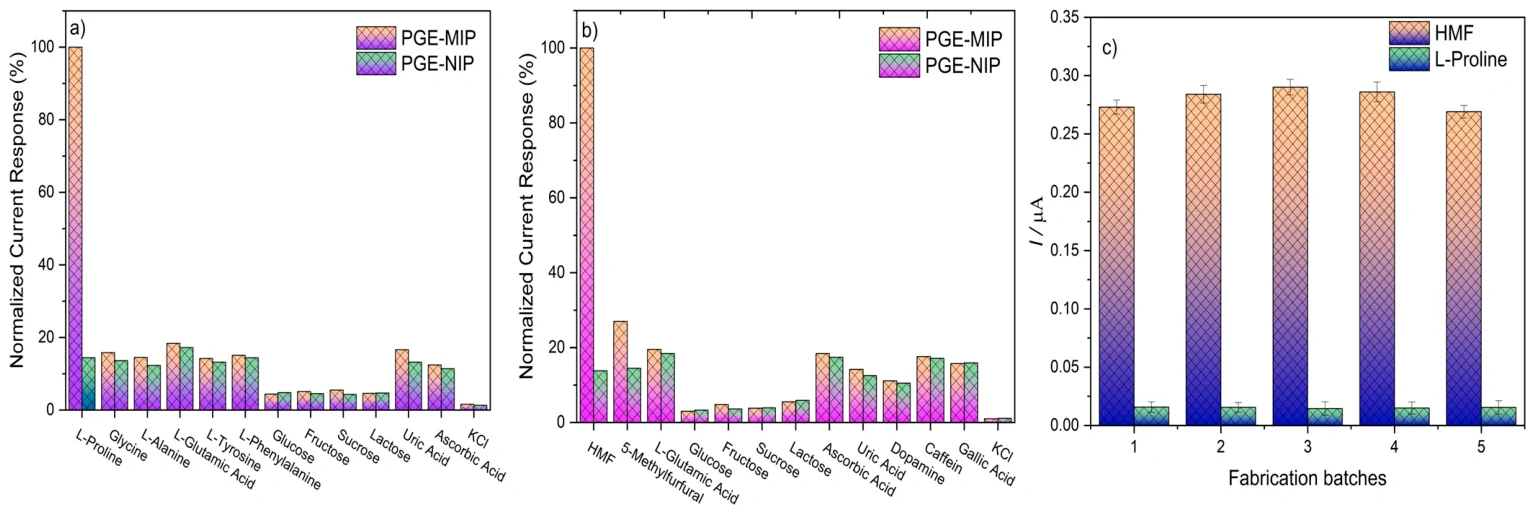
(**a**) Normalized current responses of PGE-MIP and PGE-NIP for HMF in the presence of various interferents. (**b**) Normalized current responses for L-Pro in the presence of various interferents. (**c**) Reproducibility of the PGE-MIP electrochemical probes for HMF and L-Pro determination.

The degree of selectivity exhibited by the MIP-based electrochemical sensors developed in this work towards L-Pro and HMF was investigated through amperometric analysis in the presence of structurally analogous compounds acting as possible interferents, each of which was present in a concentration 25 times higher than the concentration of the target molecule (Figure 10a,b). The L-Pro-imprinted sensor demonstrates a high degree of selectivity towards L-Pro, in which a tiny current response is generated in the presence of structurally analogous amino acids, sugars, organic acids, and inorganic compounds, which are barely 20% of L-Pro, despite having high levels of chemical similarities. Likewise, high responses of the HMF-imprinted sensor are dominated by HMF alone, whereas high responses of chemically analogous molecules and common electrochemically active interferents are barely a quarter of a concentration higher. These observations demonstrate that high degrees of molecular recognition are generated due to high shape and functional complementarity of the imprinting voids, efficiently inhibiting non-specific adsorptions. In fact, the respective PGE-NIP electrodes demonstrate non-discriminatory responses to both target molecules and interfering substances. Consequently, the distinct difference between the MIP and NIP responses further supports the notion that the observed selectivity stems from the molecular imprinting process rather than the inherent electroactivity of the target molecules.

On the basis of the findings displayed in Figure 9 and Figure 10, it is essential to explain imprinting and recognition mechanism in detail by taking interaction between pyrrole and the target molecules. The MIP-modified electrode exhibits a high peak current and improved sensitivity toward the target analyte in comparison with the NIP-modified electrode, as proven by Figure S2. To quantitatively express the enhancement in sensor response due to molecular imprinting, the imprinting factor (IF), defined as the ratio of the analytical signal obtained with the MIP electrode to that obtained with the corresponding NIP electrode under identical conditions (IF = IMIP/INIP), was calculated from the DPV responses shown in Figure S2 and Figure 9. The resulting IF values were 5.26 for L-Pro and 4.13 for HMF, confirming a substantially higher response on the imprinted surface and providing a standard quantitative measure of the effectiveness of the molecular imprinting process, consistent with the qualitative trend already described above. Consequently, the distinct difference between the MIP and NIP responses further supports the notion that the observed selectivity stems from the molecular imprinting process rather than the inherent electroactivity of the target molecules. This observation clearly indicates the successful formation of specific recognition cavities within the polymer matrix.

As previously shown in Figure 10, the smaller response obtained for structurally related interferents demonstrates that the generated electrochemical signal arises from selective molecular recognition rather than from non-specific surface adsorption. These findings indicate that the imprinting process leads to a significant increase in peak current due to the effective entrapment of the target molecule within the cavities, consequently boosting the analytical performance. Altogether, these data strongly indicate that specific non-covalent interactions (hydrogen bonding and electrostatic for L-Pro, and hydrogen bonding and π-π stacking for HMF) govern both the imprinting process and the subsequent selective rebinding.

In order to test the reproducibility of the designed sensing device, an experimental setup was established to measure the DPV responses of five different constructed PGE-MIP electrodes for the detection of HMF and L-Pro against 20 µM HMF or 20 µM L-Pro, tested in three parallel replicates each. The results as displayed in Figure 10c indicated very reliable measurements and low differences, reflected by a relative standard deviation (RSD) of 3.20% for HMF and 3.22% for L-Pro. The repeatability of the sensor was assessed by performing five successive DPV measurements of 25 µM HMF or 25 µM L-Pro using the same PGE-MIP electrodes separately fabricated for the sensing of HMF and L-Pro. The resulting current responses showed an RSD of 1.97% for HMF and 2.27% for L-Pro, reflecting excellent repeatability of the signal. Moreover, long term stability of the DPV signal was followed up for a period of 15 days. Although a slight decrease was recorded in the DPV responses of both sensing platforms, PGE-MIP-based HMF- and L-Pro-sensing platforms retained 81.4% and 83.1% of their original signal, respectively, confirming their suitability for long-term applications. The PGE-MIP electrodes were kept in 0.1 M PBS with a pH of 7.4 at 4 °C throughout the 15-day stability study. DPV responses were obtained at daily intervals over the 15 days. The decrease in signals was slow the entire 15 days showing that the method of storing guarantees consistent usage over time.

### 3.9. L-Pro and HMF Determination in Real Honey Samples

In the course of the experimental procedure, a quantity of one gram of honey sample was accurately measured, and 10 mL of PBS with a pH of 7.4 were added to it. DPV measurements were then obtained. Scans were conducted within the range of 0 to 1.7 V for L-Pro and 0 to −1.5 V for HMF. Each measurement was repeated thrice. The data obtained from this study are presented in Table 2. All electrochemical measurements were carried out in 0.1 M PBS (pH 7.4) at room temperature (20 °C). The buffer solution was purged with high-purity N_2_ for 5 min before measurements to eliminate the influence of dissolved oxygen. The CV, DPV and CA detection was performed versus Ag/AgCl.

Prior to the thermal treatments, in the untreated state at ambient temperature, the sensor measured the concentration of L-Pro from 540.13 μM (Sample 1) to 4275.86 μM (Sample 3) for the six samples of honey (Table 2)—a nearly eightfold difference in line with expected compositional differences in commercial, locally-produced, and monofloral honeys of different botanic origins. Precision was excellent for all untreated samples, with RSDs of 2.40% to 4.78% (*n* = 3), which fall well within the usual range of acceptable precision criteria for analytical procedures. Thus, the findings confirm the ability of the PGE-MIP-PPy sensor to quantify L-Pro directly in the honey matrix. At room temperature, HMF was present in four out of the six honey samples at concentrations between 8.54 and 12.31 μM (Table 2), while in Samples 3 and 4, HMF was not detected because of the absence or extremely low concentration of HMF in fresh honey. All of the results for HMF detection in all samples under the various conditions by DPV were very close to the results obtained using the HPLC and UV reference methods with the mean absolute relative deviation of 3.8% (0.5–7.6%, *n* = 16).

### 3.10. Temperature Effect

Table 2 summarizes the effect of controlled thermal treatment (70 °C and 90 °C, 2 h) on the L-Pro and HMF content of the six honey samples. Across all samples, heating produced a consistent and inverse trend: L-Pro concentration decreased progressively with increasing temperature, while HMF concentration increased correspondingly, reaching its highest value at 90 °C in every sample tested. This concurrent, opposite-direction response is consistent with the established roles of the two markers, in which proline reflects botanical maturity and authenticity while HMF reflects heat exposure and storage-related degradation; observing both markers move together in the expected directions therefore provides converging, rather than independent, evidence of thermally induced quality loss. From the data presented in the Tables and Figures, it can be seen that the extent of the decline in L-Pro concentration was not directly proportional to the extent of the rise in HMF concentration in all samples tested. For example, Sample 1 demonstrated a relatively large L-Pro loss compared to a relatively small HMF gain. On the other hand, Sample 4 showed a relatively low L-Pro loss along with a higher HMF gain at 70–90 °C. Such behavior of HMF and L-Pro changes is to be expected due to the fact that both the depletion of L-Pro and production of HMF are driven by different pathways, which have only partial overlap. Namely, in addition to producing HMF, L-Pro is involved in several different Maillard reaction pathways, while the production of HMF is driven by sugar dehydration, followed by decomposition of the compound when it is subjected to heating. The degree of their interaction depends on several honey-specific factors: the composition and ratio of the initial sugar content, moisture, pH and mineral/enzyme content. These factors vary between botanical sources and likely account for the observed sample-to-sample differences. It is therefore interpreted that the consistent directional (inverse) relationship between L-Pro and HMF across all samples, rather than a fixed quantitative ratio between them, is the chemically meaningful and expected outcome.

Mechanistically, the increase in HMF is attributable to the acid-catalyzed dehydration of hexose sugars (fructose and glucose), a reaction that is strongly accelerated by heat and, to a lesser extent, by prolonged storage and elevated acidity [4,8]. As HMF is virtually absent in fresh, unprocessed honey, its accumulation during heating serves as a direct chemical signature of thermal stress.

The parallel decrease in free L-Pro is best explained by its consumption as a reactant in the Maillard reaction. As a secondary amine, proline reacts readily with the reducing sugars released during heating to form Amadori rearrangement products, which subsequently condense further to yield melanoidins [7,53]. This pathway progressively converts free, electrochemically active L-Pro into larger, electro-inactive condensation products, thereby lowering the free L-Pro signal measured by the sensor. Because sugar dehydration (which forms HMF) and amino acid consumption (which depletes L-Pro) are coupled steps of the same Maillard cascade, the two measurements together generate a combined electro-chemical fingerprint of thermal degradation in honey [54].

To verify the reliability of sensor-based HMF and L-Pro measurements results were cross-validated against reference HPLC and UV methods for HMF and L-Pro, respectively. The HMF and L-Pro values obtained with the PGE-MIP-PPy sensor notably matched those obtained by HPLC and UV across all samples and temperatures, with considerably lower RSD values. Taken together, these results demonstrate that the developed MIP-based sensor platform reliably tracks the coupled evolution of L-Pro depletion and HMF formation during thermal processing, supporting its use as a practical tool for monitoring honey quality under heat exposure.

In order to examine whether the correlation between the results of the sensor and reference methods is statistically significant, the paired data of HMF (sensor versus HPLC) and L-Pro (sensor versus UV–Vis spectrophotometric method) were analyzed using paired t-test and Pearson’s correlation. For HMF, the paired t-test gave t = 0.17, *p* = 0.87, which means that there is no statistically significant difference between the two methods, and there is also a strong correlation (Pearson’s r = 0.98, *p* < 0.0001; linear regression slope = 1.11, R^2^ = 0.97). In the case of L-Pro, the paired t-test gave t = 2.86, *p* = 0.011, which means that there is a small, yet statistically significant systematic bias (bias = 4.68 μM; absolute relative deviation = 0.51%, *n* = 18) between the two methods, whereas the correlation between the two methods is perfect (Pearson’s r = 0.99, *p* < 0.0001; linear regression slope = 0.99, R^2^ = 0.99). Due to the high precision of both methods, the statistically significant t-test result for L-Pro means that there is a small systematic offset of less than 1%, while the near-unity slope and R^2^ of linear regression demonstrate that the sensor readings follow the reference method results correctly.

Furthermore, the compliance of individual thermally treated samples with the regulatory honey-quality criteria was rigorously evaluated based on the established mass concentration framework, incorporating the experimental data compiled in Table 2. According to international food standards and the Turkish Food Codex, the HMF concentration must not exceed the threshold of 40 mg/kg, which corresponds strictly to 317.2 µmol/kg on a direct raw honey mass basis. The quantitative monitoring presented in Table 2 demonstrated a progressive, temperature-dependent increase in HMF content across the heated samples. Crucially, even after the most severe thermal treatment at 90 °C, the maximum detected HMF concentration among all individual honey samples reached only 22.73 µmol/kg (with a corresponding RSD of 3.03%). This peak value, as well as the lower temperature profiles evaluated at 70 °C (where the maximum HMF content was found to be 14.78 µmol/kg), remains safely and substantially below the statutory 317.2 µmol/kg threshold. These findings confirm that the analytical results, expressed directly per kilogram of honey, validly demonstrate the baseline freshness and quality of the evaluated samples under all investigated heating conditions, while successfully addressing the regulatory compliance tracking capability of the developed sensor.

It was determined that the analytical results, expressed per kilogram of honey under the experimental conditions examined, indicated that the honey still retained its basic freshness and quality levels, and it was demonstrated that the sensor developed successfully demonstrated its ability to monitor compliance with regulations. Whilst the HMF levels in the samples did not exceed the legal limit under these conditions, it should also be borne in mind that HMF levels may increase depending on the degree and duration of heating or storage conditions.

## 4. Discussion

Threshold criteria defining the parameters of the authentic honey sample have been established internationally. Thus, according to the European Union and Codex Alimentarius Commission, there is a maximum permissible concentration of HMF equal to 40 mg/kg (317 µM) and a minimum required concentration of L-Pro equal to 300 mg/kg (2606 µM) [5,6]. In terms of a mass-equal molar concentration and using the molecular weights of HMF (126.11 g/mol) and L-Pro (115.13 g/mol), respectively, these thresholds amount to approximately 317 μM (maximum threshold of HMF) and 2606 μM (minimum threshold of L-Pro).

Since both measurements lie within the range or near to the working range of the developed sensors (10–1600 μM for both analytes), this means that the two developed sensing platforms meet the requirements set by the regulatory authorities and are applicable for use in screening.

Comparative assessment of the performance of the developed PGE-MIP-PPy sensors in terms of analytical figures of merit, relative to previously reported electrochemical methods, is given in Table 1. The lowest LOD for the determination of HMF was reported to be 0.048 μM in the case of the square wave voltammetry method by Salhi et al. [45]; however, the highest value of LOD was reported by Oliveira et al. [43] to be equal to 180 μM. The LOD of the present study (4.81 μM) is significantly better than the latter one (≈37 times), although being nearly equal to the LOD of Salis et al. [9] (0.6 ppm ≈ 4.76 μM), who report on a bare-electrode SWVmethod lacking molecular recognition elements. At the same time, it does not reach the sub-micromolar detection limit of the screen-printed electrode of Santos et al. [28] (0.068 μM) and the nanomaterial enhanced platforms of Salhi et al. [45], Hou et al. [47], Cutaia et al. [49] and Zhang et al. [51] (0.048–2.13 μM). They use strategies specifically aimed at improving electron transfer efficiency and effective surface area, such as gold nanoparticle-doped graphene hydrogel [51], bioelectrocatalytic amplification [47] or higher surface area industrial platform of screen-printed carbon electrodes [28,49]. Our sensor design does not include any of these.

It needs to be mentioned that the PPy electropolymerization and over-oxidation-based template removal are known methods, as can be seen from our previous works on various target molecules [30,33,34]. Those methods are not introduced as novel methods in this work. The novelty of the present work lies elsewhere, such as: creation of two different single-analyte MIP-PPy/PGE sensors for L-Pro and HMF based on one and the same inexpensive fabrication technique and not on two different designs; optimization of electro-polymerization conditions not screened before for such analyte pairs; and direct application of the technique to honey without performing liquid–liquid extraction in contrast to the method of Francisco et al. [24] employing the SALLE technique.

The sensor described by Cutaia et al. [49] is, as far as we know, the methodologically closest comparator in Table 1 in that both sensors use a molecularly imprinted polypyrrole film to detect furaldehyde in honey; the lower LOD (0.8 μM vs. 4.81 μM) of the former is likely due to its use of screen-printed carbon as opposed to a hand-cut pencil graphite lead, not due to the imprinting chemistry.

In the case of L-Pro, Table 1 clearly shows a significantly widened range of LODs. Nanomolar to sub-nanomolar LODs were demonstrated by enzymatic biosensors and biosensors utilizing nanomaterial and optical amplification, such as proline dehydrogenase immobilized on mesoporous silica, graphene-quantum-dot/gold-nanoparticle composites, and electrochemiluminescence. In comparison, the two enzymatic biosensors in Table 1 without nanomaterial or optical amplification, based on direct enzyme amperometric or chronoamperometric transduction, achieve much higher LODs of 0.05 mM (50 μM [10]) and 0.68 mM (680 μM [44]) that are one to two orders of magnitude inferior to the LOD in this study. This indicates the advantage of molecular imprinting in comparison with unamplified enzyme transduction for this analyte, even though the sensitivity of molecular imprinting is inferior to that of catalytically or nanomaterial-amplified platforms, as well as to that of the non-enzymatic MIP configurations developed for the determination of free proline in botanical extracts. It is important to note that the discrepancy in LOD sensitivity should be considered along with the concentrations that are actually relevant to the analysis of honey samples. As can be seen from Table 2, the initial concentration of L-Pro in the analyzed honey samples varies in the range of 540 to >4000 μM and remains above 500 μM in almost all samples even after thermal degradation—several orders of magnitude higher than both the LOD of the present method (5.39 μM) and the regulation limits. Also, the concentration of HMF in honey samples (8–23 μM before thermal degradation) remains significantly higher than the 4.81 μM LOD. The increased sensitivity enabled by enzyme/nanomaterial-based amplification schemes, without a doubt, is extremely useful for trace clinical and environmental analysis, but is excessive for the analysis of honey authenticity and freshness screening. In this case, the analytically interesting concentrations are of micromolar/millimolar order of magnitude. Thus, in the above-discussed context, the LOD of the present sensor positioned roughly two to three orders of magnitude lower than both the real sample concentrations and regulation limits is sufficient for its intended application, and the sensitivity sacrifice compared to the most advanced techniques in Table 1, should be traded off against practical advantages outlined further.

The present research differs from the overwhelming majority of the methods listed in Table 1 in terms of three practical aspects. First of all, the method does not rely on the use of biological recognition elements (enzymes, antibodies), and engineered nanomaterials (e.g., gold nanoparticles, graphene, quantum dots). The use of such materials, generally, implies additional synthesis or immobilization steps, need for storage, and batch-to-batch variations [6,10,44,46,48,50,51]. In contrast, the present PPy-based MIP layer is synthesized in one step via electropolymerization on a disposable, inexpensive pencil graphite electrode.

Secondly, whereas most of the previous literature discussed in Table 1 focuses separately on HMF [9,28,43,45,47,49,51] or L-Pro [6,10,44,46,48,50,52], the current work shows how both these analytes can be detected using the same electrode fabrication platform, where they just use different template molecules. This enables combined authenticity (L-Pro) and freshness (HMF) assessment from a single low-cost electrode design, a combination not previously reported for honey analysis, to the best of our knowledge. Thirdly, in contrast to the SALLE-assisted approach of Francisco et al. [24], which necessitated a separate liquid–liquid extraction step prior to measurement, the present method is applied directly to a simple aqueous dilution of the honey sample, thereby simplifying the analytical work flow.

Finally, direct cross-study comparison should be made with some caution: several of the L-Pro sensors in Table 1 were developed and validated in biological fluids (plasma, serum) [6,46,50] or plant tissue [52] rather than honey, and the enantioselective sensor of Wang et al. [29] (not listed in Table 1, but discussed in the Introduction) addresses a different analytical question—chiral discrimination between proline enantiomers—rather than total proline quantification. The composition of the sample matrix exerts a significant influence on the achievable sensitivity and selectivity. Consequently, absolute LOD values across matrices are indicative rather than strictly equivalent. In conclusion, the PGE-MIP-PPy sensors developed in this study do not attain the lowest absolute LOD reported for either analyte in Table 1. However, they are sensitive enough to screen honey quality according to both regulatory limits and concentrations of real samples. Furthermore, they are also more straightforward and more cost-effective to fabricate compared to the enzyme or nanomaterial based counterparts. A notable aspect of this study is that it proposes a common sensor fabrication design, separately applied to build two single-analyte sensors covering both quality markers, and thus demonstrates their practical significance for routine honey authenticity and freshness testing.

## 5. Conclusions

This work reports an electrochemical platform—one for L-Pro and one for HMF, the main authenticity and freshness markers of honey—built via a single, disposable, low-cost fabrication route, with only the imprinting template differing between the two, separately fabricated and operated sensors.

The electropolymerization and over-oxidation chemistry underlying these sensors are well-established techniques. The novel aspect of this work is its application to a single, shared, low-cost fabrication platform covering two complementary honey-quality markers and parametric optimization. The study results go beyond the addition of dual-marker capability, to a systematic parametric optimization of monomer-to-template ratio, electropolymerization cycle number and scan rate, and template removal conditions for this particular analyte pair. Such a combination of optimization has not been reported before for MIP-PPy-based honey sensors. The sensor-derived concentrations showed strong agreement with the corresponding reference methods: HMF measurements did not differ significantly from the HPLC results (*p* = 0.87); (r = 0.98), whereas L-Pro measurements showed a small but statistically significant systematic bias relative to the UV–Vis reference method (*p*= 0.011); absolute relative deviation (0.51%), while maintaining a strong correlation (r = 0.99). Applied to real honey samples under controlled thermal stress, the sensors correctly tracked the expected inverse evolution of L-Pro and HMF with increasing temperature, consistent with the established role of proline as a Maillard-reaction substrate and HMF as its degradation product. We regard this agreement as evidence that the developed platform faithfully captures chemically meaningful changes in the honey matrix, supporting its reliability for quality screening rather than as a novel chemical finding in its own right, since the underlying relationship is already well documented. Compared with previously reported HMF and L-Pro electrochemical sensors, which typically rely on enzymes, nanomaterials, or an auxiliary extraction step and each address only one of the two markers, the present platform trades a degree of absolute sensitivity for simplicity, low cost, direct matrix compatibility without pretreatment, and, uniquely, coverage of both quality markers through two separate, single-analyte sensors built from a common, shared fabrication process. We believe this combination is of particular value for routine, on-site, or resource-limited testing contexts where chromatographic or enzyme-based methods are impractical.

Several directions remain for future work. The present validation involved a limited set of six honey samples from three botanical origins; broader validation across a larger number of samples, additional botanical and geographical origins, and inter-laboratory comparison would strengthen confidence in the method’s general applicability. The dual-sensor format also invites exploration of a combined L-Pro/HMF response index as a single composite authenticity-freshness score, and further miniaturization to move the platform from bench-scale demonstration toward field-deployable use by producers, regulators, or quality-control laboratories. Finally, given the modular nature of the imprinting strategy demonstrated here, the same fabrication approach may be extendable to other honey adulteration or quality markers beyond L-Pro and HMF, broadening the scope of application of this common, low-cost sensor fabrication approach for comprehensive honey authentication.

## Figures and Tables

**Table 1:** Comparison of the proposed method with previous literature reports for L-Pro and HMF determination.

Hmf				L-Pro			
Method	Linear Range	LOD	Ref.	Method	Linear Range	LOD	Ref.
SWV	2–200 ppm	0.6 ppm	[9]	DPV	1 µM–1 nM	1 nM	[6]
SWV	0.2–2.0 µM	0.068 µM	[28]	Amp	-	0.05 mM	[10]
LSV	0.35–11 mM	0.18 mM	[43]	ChA	2.26 to 15 mM	0.68 mM	[44]
SWV	0.06–0.28 ppm	0.006 ppm	[45]	DPV	0.01–0.15 μM	0.006 μM	[46]
ChA	0.002–0.23 mM	0.5 µM	[47]	ECL	0.1−10 µM	75 nM	[48]
SWV	0.8–20 µM	0.8 µM	[49]	DPV	0.5 nM–10 mM	0.1 nM	[50]
DPV	0.01–10 mM	2.13 µM	[51]	DPV	1 × 10^−13^–10 mM	9.18 × 10^−15^ M	[52]
DPV	10–1600 µM	4.81 µM	This work	DPV	10–1600 µM	5.39 µM	This work

ChA, chrono-amperometry; SWV, square wave voltammetry; LSV, linear sweep voltammetry; DPV, differential pulse voltammetry. Amp, amperometry; ECL, electrochemiluminescence.

**Table 2:** Effect of the thermal treatment (70 °C and 90 °C) on L-Pro and HMF concentrations (*n* = 3).

Honey Samples	Before Heat Treatment L-Proline Content (µM)/RSD %	70 °C L-Proline Content (µM)/RSD%	90 °C L-Proline Content (µM)/RSD%	Before Heat Treatment L-Proline Content (µM) with UV/RSD %	70 °C L-Proline Content (µM) with UV/RSD%	90 °C L-Proline Content (µM) with UV/RSD%	Before Heat Treatment HMF Content (µM)/RSD%	70 °C HMF Content (µM)/RSD%	90 °C HMF Content (µM)/RSD%	Before Heat Treatment HMF Content (µM) with HPLC/RSD%	70 °C HMF Content (µM) with HPLC/RSD%	90 °C HMF Content (µM) with HPLC/RSD%
Sample 1	540.13/2.40	340.01/2.17	99.29/2.10	537.23/3.25	342.16/2.75	101.13/3.73	10.40/1.44	12.91/1.88	15.69/2.35	11.25/1.43	12.45/2.32	14.77/2.05
Sample 2	1278.57/3.90	1170.43/3.45	840.86/3.58	1267.44/3.12	1165.81/2.69	837.17/4.29	8.54/1.51	14.10/1.95	15.22/2.66	9.20/1.77	15.12/1.85	15.44/2.27
Sample 3	4275.86/4.78	1692.86/3.67	644.60/3.44	4263.65/2.87	1697.49/3.53	639.10/4.17	-	14.78/1.47	15.62/1.97	-	15.34/2.31	15.88/2.89
Sample 4	2740.57/3.77	2647.14/3.73	635.71/3.86	2733.44/4.11	2641.33/3.62	634.82/3.66	-	14.02/1.37	22.73/3.03	-	13.78/1.66	21.14/2.94
Sample 5	3288.14/3.89	713.86/2.63	543.70/3.52	3271.33/2.77	711.57/4.22	545.87/4.07	11.02/1.55	14.63/2.11	18.39/2.14	10.63/1.62	14.42/1.88	17.87/2.63
Sample 6	4198.43/4.45	1464.29/3.44	888.29/3.89	4177.61/4.22	1459.91/3.66	891.39/3.27	12.31/1.48	14.76/2.24	15.65/1.91	12.07/1.71	14.84/2.08	16.10/1.75

Note: The values in Table 2 represent final, recycled concentrations (μmol/kg) calculated based on crude honey mass, rather than crude solution measurements in the cell. Method-specific sample preparation protocols—1:10 dilution (1.0 g honey in 10 mL) for DPV and HPLC and 1:20 dilution (5.0 g honey in 100 mL) for UV–Vis reference methods—were fully integrated into the calculation framework to obtain a unified and accurate benchmark.

## Data Availability

The original contributions presented in this study are included in the article/Supplementary Materials. Further inquiries can be directed to the corresponding author.

## Supplementary Materials

The following supporting information can be downloaded at: https://www.mdpi.com/article/10.3390/foods15183325/s1, Figure S1: The effect of scan rate recorded for PEG-MIP in 1 mM of (a) HMF and (c) L-Pro. Linear relationship between cathodic peak current of (b) HMF and anodic peak current of (d) L-Pro versus square root of scan rate; Figure S2 Cyclic voltammograms (7 cycles) of PGE-MIP-PPy and PGE-NIP-PPy electrodes in the presence of 400 µM L-Pro.

## References

[1] Meda A., Lamien C.E., Romito M., Millogo J., Nacoulma O.G. (2005). Determination of the total phenolic, flavonoid and proline contents in Burkina Fasan honey, as well as their radical scavenging activity. Food Chem..

[2] Truzzi C., Annibaldi A., Illuminati S., Finale C., Scarponi G. (2014). Determination of proline in honey: Comparison between official methods, optimization and validation of the analytical methodology. Food Chem..

[3] Wen Y.-Q., Zhang J., Li Y., Chen L., Zhao W., Zhou J., Jin Y. (2017). Characterization of Chinese Unifloral Honeys Based on Proline and Phenolic Content as Markers of Botanical Origin, Using Multivariate Analysis. Molecules.

[4] Al-Farsi M., Al-Belushi S., Al-Amri A., Al-Hadhrami A., Al-Rusheidi M., Al-Alawi A. (2018). Quality evaluation of Omani honey. Food Chem..

[5] Chin N.L., Sowndhararajan K., Arnaut de Toledo V.D.A., Chambó E.D. (2020). A Review on Analytical Methods for Honey Classification, Identification and Authentication. Honey Analysis—New Advances and Challenges.

[6] Hasanzadeh M., Nahar A.S., Hassanpour S., Shadjou N., Mokhtarzadeh A. (2018). Immobilization of Proline Dehydrogenase on Functionalized Silica Mesoporous Nanomaterial Towards Preparation of a Novel Thermostable Enzyme Biosensor. J. Nanosci. Nanotechnol..

[7] Wang H.-Y., Qian H., Yao W.-R. (2011). Melanoidins produced by the Maillard reaction: Structure and biological activity. Food Chem..

[8] Shapla U.M., Solayman M., Alam N., Khalil M.I., Gan S.H. (2018). 5-Hydroxymethylfurfural (HMF) levels in honey and other food products: Effects on bees and human health. Chem. Cent. J..

[9] Salis S., Spano N., Ciulu M., Floris I., Pilo M.I., Sanna G. (2021). Electrochemical Determination of the “Furanic Index” in Honey. Molecules.

[10] Zheng H., Lin L., Okezaki Y., Kawakami R., Sakuraba H., Ohshima T., Takagi K., Suye S.-I. (2010). Electrochemical behavior of dye-linked L-proline dehydrogenase on glassy carbon electrodes modified by multi-walled carbon nanotubes. Beilstein J. Nanotechnol..

[11] Jöbstl D., Husøy T., Alexander J., Bjellaas T., Leitner E., Murkovic M. (2010). Analysis of 5-hydroxymethyl-2-furoic acid (HMFA) the main metabolite of alimentary 5-hydroxymethyl-2-furfural (HMF) with HPLC and GC in urine. Food Chem..

[12] Reyes-Salas E.O., Manzanilla-Cano J.A., Barceló-Quintal M.H., Juárez-Mendoza D., Reyes-Salas M. (2006). Direct Electrochemical Determination of Hydroxymethylfurfural (HMF) and its Application to Honey Samples. Anal. Lett..

[13] Tang N., Wei Y., He Q., Shi S., Zhou C., Chen A., Liu J. (2024). Facile electrochemical determination of sertraline at nanomolar concentration using bimetallic MXene NbTiCTx and MWCNTs hybridized electrodes as sensing platform. Mater. Today Chem..

[14] Beykaya M. (2021). Determination of physiochemical properties of raw honey samples. Prog. Nutr..

[15] Singh R., Gupta R., Bansal D., Bhateria R., Sharma M. (2024). A Review on Recent Trends and Future Developments in Electrochemical Sensing. ACS Omega.

[16] Ilager D., Shanbhag M.M., Malode S.J., Kalanur S.S., Pollet B.G., Aleid A., Alodhayb A.N., Shetti N.P. (2025). High-Efficiency Hydrated Tungsten Oxide Nanostructures for Improved Sensitivity in Detecting Fungicides. J. Electrochem. Soc..

[17] Rodrigues P.N., Mascarenhas R.J., Malode S.J., Al-Otaibi J.S., Alodhayb A.N., Shetti N.P. (2025). Biowaste-Derived Activated Carbon-Based Sensor for the Electrochemical Analysis of Hesperetin and Co-Existing Flavonoids in the Presence of Triton X-100. J. Electrochem. Soc..

[18] Wu Y., Deng P., Tian Y., Feng J., Xiao J., Li J., Liu J., Li G., He Q. (2020). Simultaneous and sensitive determination of ascorbic acid, dopamine and uric acid via an electrochemical sensor based on PVP-graphene composite. J. Nanobiotechnol..

[19] Jiang Y., Sima Y., Liu L., Zhou C., Shi S., Wan K., Chen A., Tang N., He Q., Liu J. (2024). Research progress on portable electrochemical sensors for detection of mycotoxins in food and environmental samples. Chem. Eng. J..

[20] Liu Y., Liang Y., Yang R., Li J., Qu L. (2019). A highly sensitive and selective electrochemical sensor based on polydopamine functionalized graphene and molecularly imprinted polymer for the 2,4-dichlorophenol recognition and detection. Talanta.

[21] Malode S.J., Prabhu K., Pandiaraj S., Alodhayb A.N., Shetti N.P. (2024). Development of a 1D-WO3 and CuO nanocomposite modified electrode for enhanced detection of 2,4-dichlorophenol. Surf. Interfaces.

[22] Lahcen A.A., Saidi K., Amine A. (2025). A critical review of electrosynthesized molecularly imprinted polymers in electrochemical sensing: Pros and cons. Curr. Opin. Electrochem..

[23] Ayerdurai V., Cieplak M., Kutner W. (2023). Molecularly imprinted polymer-based electrochemical sensors for food contaminants determination. TrAC Trends Anal. Chem..

[24] Francisco K.C., Lobato A., Tasić N., Cardoso A.A., Gonçalves L.M. (2022). Determination of 5-hydroxymethylfurfural using an electropolymerized molecularly imprinted polymer in combination with Salle. Talanta.

[25] Turan H.E., Medetalibeyoglu H., Polat I., Yola B.B., Atar N., Yola M.L. (2023). Graphene quantum dots incorporated NiAl_2_O_4_ nanocomposite based molecularly imprinted electrochemical sensor for 5-hydroxymethyl furfural detection in coffee samples. Anal. Methods.

[26] Ramanavičius S., Morkvėnaitė-Vilkončienė I., Samukaitė-Bubnienė U., Ratautaitė V., Plikusienė I., Viter R., Ramanavičius A. (2022). Electrochemically Deposited Molecularly Imprinted Polymer-Based Sensors. Sensors.

[27] Terán-Alcocer Á., Bravo-Plascencia F., Cevallos-Morillo C., Palma-Cando A. (2021). Electrochemical Sensors Based on Conducting Polymers for the Aqueous Detection of Biologically Relevant Molecules. Nanomaterials.

[28] Santos R.A.D.S., Schaffel I.D.F., dos Santos G.F.S., Rodrigues J.G.A., de Q. Ferreira R.d.Q. (2025). Voltammetric determination of hydroxymethylfurfural in honey using screen-printed carbon electrodes: Optimization and in-house validation tests. J. Solid State Electrochem..

[29] Wang Y., Han Q., Han R., He K. (2017). Enantioselective Recognition of Proline Enantiomers Using Sulfhydryl-modified Self-assembled Gold Electrodes. Anal. Lett..

[30] Gürler B., Özkorucuklu S.P., Kır E. (2013). Voltammetric behavior and determination of doxycycline in pharmaceuticals at molecularly imprinted and non-imprinted overoxidized polypyrrole electrodes. J. Pharm. Biomed. Anal..

[31] Moulaee K., Neri G. (2021). Electrochemical Amino Acid Sensing: A Review on Challenges and Achievements. Biosensors.

[32] Sood Y., Singh K., Mudila H., Lokhande P., Singh L., Kumar D., Kumar A., Mubarak N.M., Dehghani M.H. (2024). Insights into properties, synthesis and emerging applications of polypyrrole-based composites, and future prospective: A review. Heliyon.

[33] Akyüz B.G., Özkorucuklu S.P., Kır E. (2021). Investigation of Electrohemical Behaviors of Some Tetracyclines Using Modified Graphite Electrodes. Süleyman Demirel Univ. J. Nat. Appl. Sci..

[34] Akyüz B.G., Özkorucuklu S.P., Kır E., Baştemur G.Y. (2020). Determination of Ciprofloxacin in Pharmaceutical Dosage, Human Serum and Urine, Using Molecularly Imprinted Polymer Modified Electrode by Voltammetry. Eur. J. Sci. Technol..

[35] Wu Y., Li G., Tian Y., Feng J., Xiao J., Liu J., Liu X., He Q. (2021). Electropolymerization of molecularly imprinted polypyrrole film on multiwalled carbon nanotube surface for highly selective and stable determination of carcinogenic amaranth. J. Electroanal. Chem..

[36] Zidarič T., Majer D., Maver T., Finšgar M., Maver U. (2023). The development of an electropolymerized, molecularly imprinted polymer (MIP) sensor for insulin determination using single-drop analysis. Analyst.

[37] Merli D., Cutaia A., Prosperi S., Zanoni C., Alberti G. (2025). Molecularly imprinted polypyrrole-modified screen-printed electrode: Toward a sensor for furfural derivatives determination. Sens. Actuators Rep..

[38] Domínguez-Renedo O., Navarro-Cuñado M.A. (2020). Molecularly imprinted polypyrrole based electrochemical sensor for selective determination of 4-ethylphenol. Talanta.

[39] Chen M., Fu Y., Cui X., Wang L., Li M. (2009). Comparative Analysis of Proline Enantiomer in Chiral Recognition of Biological Macromolecule in Immunoassay. Electroanalysis.

[40] Hemed N.M., Leal-Ortiz S., Zhao E.T., Melosh N.A. (2023). On-Demand, Reversible, Ultrasensitive Polymer Membrane Based on Molecular Imprinting Polymer. ACS Nano.

[41] Liu L., Zhang D., Jin Z., Zhang Z., Li S., Cang J. (2017). Development of an Electrochemical Approach for Proline Content Detection in Winter Wheat. Int. J. Electrochem. Sci..

[42] Ayankojo A.G., Reut J., Syritski V. (2024). Electrochemically synthe sized MIP sensors: Applications in healthcare diagnostics. Biosensors.

[43] Oliveira P.R., Guterres E Silva L.R., Kalinke C., Ferrari A.G.M., Prakash J., Wen Y., Nocelli R.C.F., Bonacin J.A., Banks C.E., Janegitz B.C. (2023). Conductive Biofilm Propolis Based: Electrochemical Determination of Hydroxymethylfurfural in Honey. Food Anal. Methods.

[44] Mirzaie A., Saadati A., Hassanpour S., Hasanzadeh M., Siahi-Shadbad M., Jouyban A. (2019). Determination of proline in human plasma samples using encapsulation of proline dehydrogenase enzyme in dendritic silica: A new platform for the enzymatic biosensing of amino acids. Anal. Methods.

[45] Salhi I., Samet Y., Trabelsi M. (2020). Direct electrochemical determination of very low levels of 5-hydroxymethyl furfural in natural honey by cyclic and square wave voltametric techniques. J. Electroanal. Chem..

[46] Hasanzadeh M., Nahar A.S., Hassanpour S., Shadjou N., Mokhtarzadeh A., Mohammadi J. (2017). Proline dehydrogenase-entrapped mesoporous magnetic silica nanomaterial for electrochemical biosensing of L-proline in biological fluids. Enzym. Microb. Technol..

[47] Hou Z., Zhang M., Wang Q., Chang L., Su H., Guo Z., Zhang L. (2025). A bioelectrocatalytic platform for 5-hydroxymethylfurfural sensing in honey. Electrochim. Acta.

[48] Kim E., Chen C., Chu M.J., Hamstra M.F., Bentley W.E., Payne G.F. (2024). Proline-Selective Electrochemiluminescence Detecting a Single Amino Acid Variation Between A1 and A2 β-Casein Containing Milks. Adv. Sci..

[49] Cutaia A., Prosperi S., Zanoni C., Alberti G. (2025). Molecularly imprinted polypyrrole based electrochemical sensor for furaldehydes determination in Italian honey samples. Sci. Rep..

[50] Hasanzadeh M., Baghban H.N., Shadjou N. (2018). Non-enzymatic Determination of L-Proline Amino Acid in Unprocessed Human Plasma Sample Using Hybrid of Graphene Quantum Dots Decorated with Gold Nanoparticles and Poly Cysteine: A Novel Signal Amplification Strategy. Anal. Sci..

[51] Zhang C., Zhang H., Wang S., Li X., Lu Y., Xu Y., Yin J., Sun S., Li J., Cao X. (2025). Direct detection of 5-hydroxymethyfurfural using a gold-nanoparticles doped graphene-hydrogel based electrochemical sensor. Microchem. J..

[52] Yan L., Luo B., Wang C., Dong H., Wang X., Hou P., Liu K., Li A. (2024). Molecularly Imprinted Polymer-Based Electrochemical Sensor for In Situ Detection of Free Proline in Cucumber Leaves. ChemElectroChem.

[53] Shahidi F. (2000). Antioxidants in food and food antioxidants. Food/Nahrung.

[54] Machado De-Melo A.A., Almeida-Muradian L.B.d., Sancho M.T., Pascual-Maté A. (2018). Composition and properties of Apis mellifera honey: A review. J. Apic. Res..

