# Supplementary material for: Rapid Assessment of Honey Quality and Authenticity via Electrochemical Determination of L-Proline and 5-Hydroxymethyl-2-Furfural Using Modified Pencil Graphite Sensors
Source: Foods. 2026 Sep 19;15(18):3325. doi: 10.3390/foods15183325 (PMC13606732; doi:10.3390/foods15183325)
Supplement: Supplementary file 1 [file foods-15-03325-s001.zip › foods-4484126-supplementary.pdf]

# Rapid Assessment of Honey Quality and Authenticity via Electrochemical Determination of L-Proline and 5-Hydroxymethyl-2-furfural Using Modified Pencil Graphite Sensors

Berrin Gürler Akyüz*[1], İzzet Koçak[1],

[1] Department of Basic Pharmaceutical Sciences, Faculty of Pharmacy, Zonguldak Bülent Ecevit University, Ibn-i Sina Campus, Esenköy, Kozlu, Zonguldak 67600, Türkiye

*Correspondence: bgurler@beun.edu.tr

Contrubuting author: izzetkocak@beun.edu.tr

## SUPPORTING INFORMATION

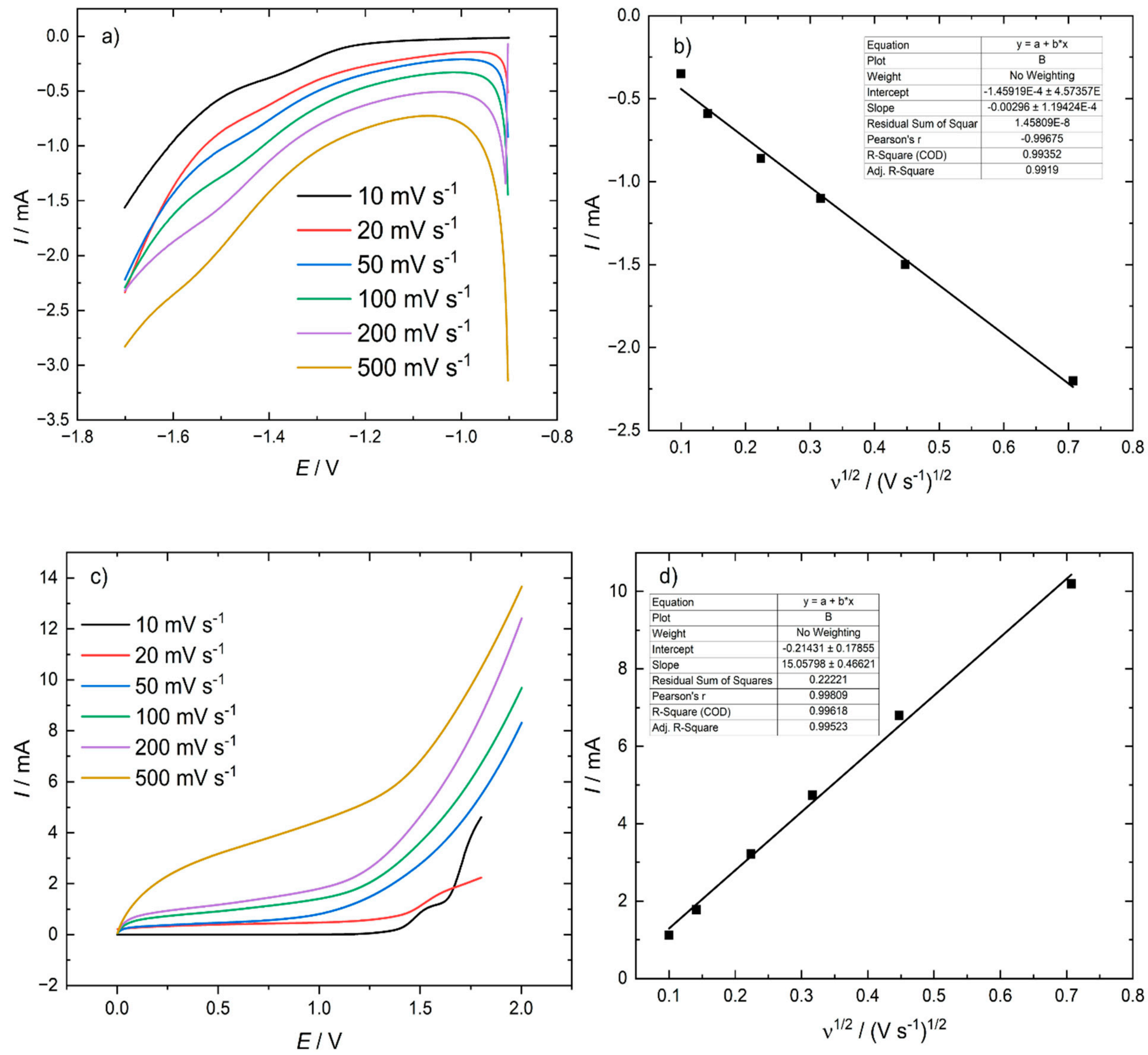


**Figure S1** The effect of scan rate recorded for PEG-MIP in 1 mM of (a) HMF and (c) L-Pro. Lineer relationship between cathodic peak current of (b) HMF and anodic peak current of (d) L-Pro versus square root of scan rate.

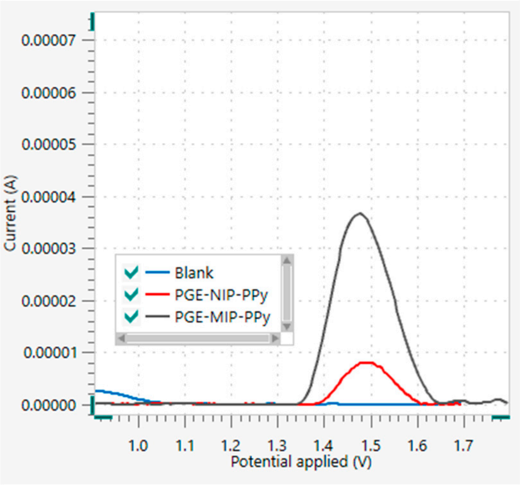


**FigureS2** Cyclic voltammograms (7 cycles) of PGE-MIP-PPy and PGE-NIP-PPy electrodes in the presence of 400 µM L-Pro.
